# Identification of enzymatic genes with the potential to reduce biomass recalcitrance through lignin manipulation in *Arabidopsis*

**DOI:** 10.1186/s13068-020-01736-6

**Published:** 2020-05-29

**Authors:** Shingo Sakamoto, Naofumi Kamimura, Yosuke Tokue, Miyuki T. Nakata, Masanobu Yamamoto, Shi Hu, Eiji Masai, Nobutaka Mitsuda, Shinya Kajita

**Affiliations:** 1 Plant Gene Regulation Research Group, Bioproduction Research Institute, National Institute of Advanced Industrial Science and Technology (AIST), Tsukuba, Ibaraki 305-8566 Japan; 2 Department of Bioengineering, Nagaoka University of Technology, Nagaoka, Niigata 940-2188 Japan; 3 Graduate School of Bio-Applications and Systems Engineering, Tokyo University of Agriculture and Technology, Koganei, Tokyo 184-8588 Japan; 4 Present Address: Graduate School of Biological Sciences, Nara Institute of Science and Technology, Ikoma, Nara 630-0192 Japan

**Keywords:** Biomass recalcitrance, High-throughput screening, Histochemical staining, Lignin, Saccharification

## Abstract

**Background:**

During the chemical and biochemical decomposition of lignocellulosic biomasses, lignin is highly recalcitrant. Genetic transformation of plants to qualitatively and/or quantitatively modify lignin may reduce these recalcitrant properties. Efficient discovery of genes to achieve lignin manipulation is thus required.

**Results:**

To screen for new genes to reduce lignin recalcitrance, we heterologously expressed 50 enzymatic genes under the control of a cinnamate 4-hydroxylase (*C4H*) gene promoter, derived from a hybrid aspen, which is preferentially active in tissues with lignified cell walls in *Arabidopsis* plants. These genes encode enzymes that act on metabolites in shikimate, general phenylpropanoid, flavonoid, or monolignol biosynthetic pathways. Among these genes, 30, 18, and 2 originated from plants, bacteria, and fungi, respectively. In our first screening step, 296 independent transgenic plants (T_1_ generation) harboring single or multiple transgenes were generated from pools of seven *Agrobacterium* strains used for conventional floral-dip transformation. Wiesner and Mäule staining patterns in the stems of the resultant plants revealed seven and nine plants with apparent abnormalities in the two respective staining analyses. According to genomic PCR and subsequent direct sequencing, each of these 16 plants possessed a gene encoding either coniferaldehyde dehydrogenase (*calB*), feruloyl-CoA 6′-hydroxylase (*F6H1*), hydroxycinnamoyl-CoA hydratase/lyase (*couA*), or ferulate 5-hydroxylase (*F5H*), with one transgenic plant carrying both *calB* and *F6H1*. The effects of these genes on lignin manipulation were confirmed in individually re-created T_1_ transgenic *Arabidopsis* plants. While no difference in lignin content was detected in the transgenic lines compared with the wild type, lignin monomeric composition was changed in the transgenic lines. The observed compositional change in the transgenic plants carrying *calB*, *couA*, and *F5H* led to improved sugar release from cell walls after alkaline pretreatment.

**Conclusions:**

Simple colorimetric characterization of stem lignin is useful for simultaneous screening of many genes with the potential to reduce lignin recalcitrance. In addition to *F5H*, the positive control, we identified three enzyme-coding genes that can function as genetic tools for lignin manipulation. Two of these genes (*calB* and *couA*) accelerate sugar release from transgenic lignocelluloses.

## Background

**Fig. 1:**
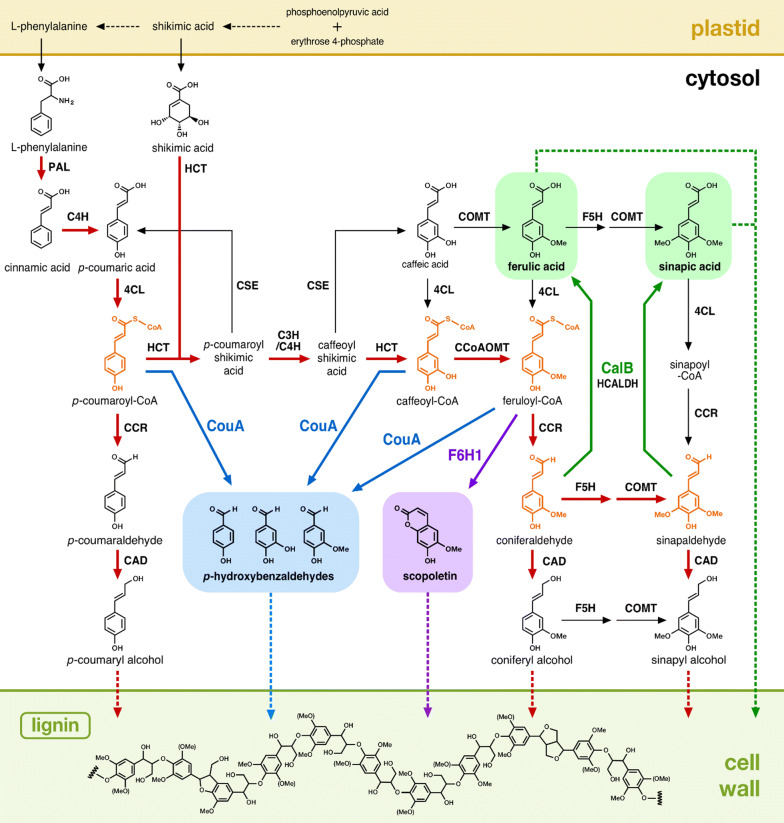
Schematic diagram of the general lignin biosynthesis pathway. The predominant route for the biosynthesis of three main monolignols is shown by red arrows. Dashed arrows indicate multiple metabolic steps. PAL, phenylalanine ammonia lyase; C4H, cinnamate 4-hydroxylase; 4CL, *p*-coumarate: CoA ligase; HCT, hydroxycinnamoyl-CoA: shikimate hydroxycinnamoyl transferase; C3H, p-coumarate 3-hydroxylase; CSE, caffeoyl shikimate esterase; CCoAOMT, caffeoyl-CoA *O*-methyltransferase; CCR, cinnamoyl-CoA reductase; F5H, ferulate 5-hydroxylase; COMT, caffeic acid *O*-methyltransferase; CAD, cinnamyl alcohol dehydrogenase; HCALDH, hydroxycinnamaldehyde dehydrogenase. The substrates expected to be converted by couA, calB, or F6H1 are shown in orange

Lignin, a phenolic and hydrophobic polymer deposited in plant cell walls, confers rigidity to cell walls and plant bodies, facilitates water transport in tissues and organs, and protects plants against biotic and abiotic stresses [1–3]. Lignin is composed of three main monolignols: *p*-coumaryl, coniferyl, and sinapyl alcohols. After polymerization, these monolignols give rise to the three main building blocks of lignin: *p*-hydroxyphenyl (H), guaiacyl (G), and syringyl (S) units. Monolignols are biosynthesized from phenylalanine and/or tyrosine in the cytosol via general phenylpropanoid and monolignol pathways [1, 4]. After transportation to the cell wall, phenoxy radicals are generated from the monolignols by phenol oxidases, such as laccase and peroxidase, and, in contrast to other cell-wall polymers, polymerize spontaneously without the need for catalytic support (Fig. 1) [5]. Combinatorial coupling of radicals gives structural complexity to lignin, thereby supporting plant growth and development [1].

However, in the utilization of secondary cell walls, lignin is highly recalcitrant during chemical and biochemical decomposition of plant biomasses for the production of biofuel and bio-based chemicals [6, 7]. The persistence of lignin even after chemical and physicochemical pretreatments hinders the efficient biorefining of plant biomass. Molecular characteristics of lignin in biomass, such as content, composition, frequency of interunit linkages, and molecular weight, influence this recalcitrance.

As an alternative to disrupting cell wall structure through pretreatments during the biorefining process to reduce lignin recalcitrance, the manipulation of plants through genetic engineering has been attempted. In early trials utilizing this approach, genes encoding monolignol biosynthetic enzymes were upregulated or downregulated to manipulate lignin content and composition [4]. One successful example was the overexpression of a gene encoding ferulate 5-hydroxylase (F5H) in transgenic plants. A gene expression cassette in which *F5H* transcription was driven by the *C4H* promoter was introduced into an *Arabidopsis* thaliana *fah1*–*2* mutant, wild-type tobacco, and poplar to manipulate lignin composition [8–10]. *F5H* expression resulted in lignin with a high number of S units in the resultant plants without any decrease in plant growth. After hot water pretreatment, a much higher glucose yield was obtained from cell wall residues with S-enriched lignin of transgenic *Arabidopsis* compared with wild-type plants [11]. Furthermore, significant increases in pulping yield were observed when wood chips from transgenic poplar overexpressing *F5H* were compared with those from wild-type plants [12].

In addition to manipulating the expression of endogenous genes, expression of exogenous genes is effective for suppression and/or rerouting of monolignol biosynthesis. Recently, Wilkerson et al. expressed a gene encoding feruloyl-CoA monolignol transferase, which was isolated from *Chinese angelica*, in poplar and successfully increased incorporation of acylated monolignols into lignin [13]. After pretreatment with mild alkali or ionic liquid, significant improvements in saccharification rate and chemical pulping yield are evident in plants harboring the gene [14, 15]. Simultaneous expression of diketide-CoA synthase and curcumin synthase 2 originating from turmeric (*Curcuma longa*) also rerouted the monolignol biosynthetic pathway in transgenic *Arabidopsis* plants for heterologous production of curcumin [16]. The resultant curcumin could be incorporated into lignin and improved saccharification efficiency of the transgenic cell wall.

In addition to plant-derived genes, heterologously expressed genes of microorganisms are available for the manipulation of lignin structure. Eudes et al. generated transgenic *Arabidopsis* plants harboring a gene encoding hydroxycinnamoyl-CoA hydratase/lyase (HCHL) from *Pseudomonas fluorescens* [17]. HCHL, whose catalytic properties resemble those of hydroxycinnamoyl-CoA hydratase/lyase (CouA; described later), catalyzes the conversion of hydroxycinnamoyl-CoAs to their corresponding hydroxybenzaldehydes (HBAlds), such as vanillin, which are also known to be incorporated into natural lignin as pendant end units [18–20]. Expression of a chimeric *HCHL* construct increased incorporation of HBAlds into lignin, reducing in turn the molecular weight of lignin and increasing the saccharification efficiency of cell wall residues prepared from the transgenic plants [17].

To identify new genetic tools for reducing biomass recalcitrance by rerouting the monolignol biosynthetic pathway, we screened 48 expression constructs containing coding sequences of 50 enzymes (Additional file 1: Tables S1 and S2) potentially able to manipulate the lignin biosynthetic pathway. The selected enzymes could be classified into several categories, namely, enzymes to prompt biosynthesis of phenolics with chemically labile units such as cinnamate esters or amides; enzymes to inhibit radical formation through the reduction of double bonds in the side chain of monolignol intermediates or by methylation of free phenolic hydroxyl groups of monolignols; enzymes to synthesize compounds with C6-C1 structures; enzymes to introduce an additional free phenolic hydroxyl group into monolignol intermediates; and enzymes expected to reverse the metabolic flow in monolignol pathway via oxidation of monolignols or corresponding aldehydes.

These genes were expressed under the control of a promoter that is active in cells with secondary cell walls, and which is derived from the *C4H* gene of hybrid aspen [21]. We identified four genes that induced changes in staining intensity and color tone by Wiesner and Mäule reagents, and/or cell shape in stem tissues of transgenic plants: two bacterial genes encoding coniferaldehyde dehydrogenase (calB) and couA, and an *Arabidopsis* gene encoding feruloyl-CoA 6′-hydroxylase (F6H1) in addition to positive control gene ferulic acid 5-hydroxylase (F5H) from *Arabidopsis*. The enzymatic saccharification efficiency of cell wall residues from the transgenic plants carrying the two bacterial genes was improved compared with wild-type plants. Overexpression of F6H1 successfully functioned for production of coumarin and structural modification of lignin, but it could not contribute to reduce the lignin recalcitrance. Our simple procedure employing lignin staining is useful for the simultaneous screening of many genes with the potential to improve biomass characteristics.

## Methods

### Plant materials

*Arabidopsis thaliana* Columbia-0 (Col-0) plants were grown in soil under 16-h/8-h day/night (60–80 μmol^−1^ s^−1^) conditions at 22 °C. Transgenic plants were generated from 8-week-old plants by the floral-dip method [22]. Transgenic plants from harvested seeds were selected on solid Murashige–Skoog (MS) medium containing 0.8% agar with 50 mg L^−1^ kanamycin.

### Gene selection and synthesis

Enzymes acting on monolignols and their precursors as the substrates were collected by searching MetaCyc [23], KEGG [24], UniProt [25], and BRENDA [26] databases and previous reports. The genes encoding these enzymes are listed in Additional file 1: Table S1. Properties of these enzymes are shown in Additional file 1: Table S2. In addition to a positive control (F5H), 49 enzymes were selected: 29, 18, and 2 from plants, bacteria, and fungi, respectively (Additional file 1: Table S1). Coding sequences were optimized for expression in *Arabidopsis*, chemically synthesized with attL Gateway recombination sites at 5´ and 3´ flanking regions, and cloned into pUC57 or pCC1 vectors (GenScript Inc.).

### Plasmid construction

Chemically synthesized DNA fragments of candidate enzymatic genes were transferred into pDEST_PkC4H_HSP_GWB4, a binary Gateway destination vector for the expression of genes under the control of the *C4H* gene promoter (*PkC4Hpro*) cloned from hybrid aspen (*Populus sieboldii *×* P. grandidentata* Y-63) [21] and terminated by the *Arabidopsis HSP18.2* terminator [27]. This destination vector was constructed by inserting approximately 2.5 kbp of the *PkC4Hpro* region into the pDEST_HSP_GWB4 vector, the latter derived from pGWB401 [28]. The *PkC4H* promoter was amplified by PCR from the plasmid pCYP73a [21] with primers PkC4Hpro_F (5´-GACTGGAAGGATGTATTGGTTGTTG-3´) and PkC4Hpro_R_Xho (5´-TTTAAACTCGAGTATCTTGGAACTGGTTTCTTTGTC-3´). Fifty genes including *F5H* (positive control) were individually cloned into pDEST_PkC4H_HSP_GWB4. *pcaH* and *pcaG* as well as *pcaH2* and *pcaG2* were placed in each same plasmid by amplifying expression cassette of *pcaG* and *pcaH2* with primers M13F_Asc (5´-CCCTTTGGCGCGCCTCGTTGTAAAACGACGGCCAGTG-3´) and HSPter_R_Asc (5´-AATTTGGCGCGCCTTATCTTTAATCATATTCCATA-3´) and inserting the amplified fragment into AscI site of the vector carrying *pcaH* and *pcaG2*, respectively. *Agrobacterium* strains harboring individual expression constructs (a total of 48 constructs, including one for *F5H*; Additional file 1: Table S1) were cultured independently, and 2–8 *Agrobacterium* cultures were mixed into a single pool. *Arabidopsis* plants were subject to genetic transformation using the traditional floral-dip procedure with seven pools of *Agrobacterium* strains (see the column “Gene set” in Additional file 1: Table S3). After recovery of transgenic lines grown from T_1_ seeds on selective medium with kanamycin, the introduced gene(s) in each transgenic plant was confirmed by the method described below.

### GUS staining

The *GUS* gene was transferred from a pENTR-gus vector (Thermo Fisher Scientific Inc.) into the pDEST_PkC4H_HSP_GWB4 vector. Transgenic *Arabidopsis* plants were produced by the floral-dip method as described above. T_1_ transgenic plants were grown for 5 weeks, and sliced sections of the inflorescence stem were stained in X-gluc solution containing 100 mM potassium phosphate, 20% (v/v) methanol, 0.5 mM potassium hexacyanoferrate, 0.5 mM potassium ferrocyanide, and 0.05 mg mL^−1^ 5-bromo-4-chloro-3-indolyl-β-d-glucuronic acid.

### Identification of introduced genes in transgenic plants

Because several constructs were mixed for *Arabidopsis* transformation, introduced genes needed to be identified in each transgenic plant. Genomic DNA was extracted from immature leaves of each transgenic plant (T_1_) using extraction buffer [200 mM Tris–HCl (pH 8.0), 250 mM NaCl, 25 mM EDTA and 0.2% SDS] and partially purified by ethanol precipitation. The introduced genes were amplified using ExTaq polymerase (Takara Bio Inc.) with the following primers: PkC4Hpro_pd (5′-AAACCCAAGCTCTCCTCATCCTGTTGC-3´) and HSPter_R (5′-GCCACAAATTCATAACACAACAAGCCA-3´). All PCR products were separated on 0.8% agarose gel, extracted with a gel extraction kit (Promega Inc.), and sequenced.

### Preparation of cross sections and lignin staining

Transgenic plants were selected on 0.8% agar MS medium containing kanamycin for 2 weeks, transferred to soil, and grown for a further 5 weeks. Cross sections were prepared from the inflorescence stem 3 cm above the soil. For high-throughput lignin staining, several hand-sliced sections from each transgenic plant were placed into separate wells of a 96-well plate and rinsed with water several times before staining. Lignin was visualized by Wiesner and Mäule staining as previously described with some modifications [29] and observed using a SZ61 stereo microscope (Olympus Inc.). For Wiesner staining, sections were stained for 5 min with 5% (w/v) phloroglucinol dissolved in concentrated HCl, followed by replacement with 30% (v/v) HCl. For Mäule staining, sections were stained with 1% (w/v) KMnO_4_ for 5 min, followed by incubation in 10% (v/v) HCl for 5 min and replacement of the solution with 1.5 M Na_2_CO_3_. Stem segments of individual transgenic plants were mounted on a 5% agar block, and 50-μm-thick cross sections were prepared using a vibrating microtome (HM-650 V; Microm Inc.). The 50-μm-thick sections subjected to Wiesner or Mäule staining were observed under an Axioscop2 fluorescent microscope (Carl-Zeiss Inc.).

### Lignin quantification

Senesced inflorescence stem was cut into 1-cm segments and fixed in methanol overnight. After three exchanges of methanol, the solution was replaced three times with acetone, and the same procedures were followed with methanol/chloroform (1:1, v/v). The resulting stem segments were rinsed twice with ethanol and dried overnight at 65 °C. Dried stem segments were ground using a Shakemaster Neo (Biomedical Science Inc.) with a stainless-steel bead and three zirconia beads. The resulting powder was gelatinized in 0.1 M sodium malate buffer (pH 6.0) at 65 °C for 10 min, and starch was then digested with 500 U mL^−1^ α-amylase (Megazyme Inc.) and 0.33 U mL^−1^ amyloglucosidase (Megazyme Inc.) in 0.1 M sodium malate buffer (pH 6.0) at 37 °C for 18 h. The destarched powder was washed with ultrapure water, rinsed three times with 100% ethanol, and dried overnight at 65 °C. The resultant powder was regarded as the alcohol-insoluble residue (AIR) and used to determine lignin content. Lignin content in the AIR was determined by the micro-Klason method described previously [30] with some modification; instead of water, 10% ethanol was used to rinse residues. AIR powder (2.0–3.0 mg) was hydrolyzed by the two-step sulfuric acid method, and the resulting residue was weighed as the acid-insoluble residue. Levels of acid-soluble lignin were calculated based on the ultraviolet absorbance of the supernatant after acid hydrolysis at 205 nm and a lignin extinction coefficient of 110 L g^−1^ cm^−1^.

### Pyrolysis–gas chromatography/mass spectrometry (Py-GC/MS) analysis

The analysis of Py-GC/MC detection data was performed according to Van Erven et al. [31]. Pyrolysis was carried out with an EGA/PY-3030D multi-shot pyrolyzer (Frontier Laboratories Inc.) using the above-mentioned AIR powder. The pyrolyzer was connected to a gas chromatographer–mass spectrometer system (GCMS-QP2020, Shimazdu Inc.) equipped with a capillary column (30 m × 0.25 mm; 0.25 μm film thickness; Ultra ALLOY^+^-5; Frontier Laboratories Inc.). The conditions of pyrolysis, and GC and MS settings were based on those described in Van Erven et al., [31]. Pyrolysis of the AIR sample was performed at 500 °C for 2 min via the split/splitless injector on the column (at 250 °C). The split ratio was 1:30 and helium was used as carrier gas with constant flow at 1.72 ml min^−1^. The GC oven was programmed as follows: hold at 70 °C for 2 min, elevate to 270 °C at 6 °C/min, then hold at 270 °C for 10 min. MS detection was completed using the electron ionization method with 70 eV electrons. The source temperature was set to 250 °C, the scan range was set to 50-550 *m/z*, and the scan rate was 2000 scans/s. Conventional pyrolysis products were quantified using the response factors described in Van Erven et al. [31].

### Thioacidolysis monomer analysis

Thioacidolysis of plant samples was performed according to the method of Yamamura et al. [32]. Briefly, freshly made thioacidolysis reagent containing 87.5% dioxane, 10% ethanethiol (97%, Alfa Aesar Inc.), and 2.5% boron trifluoride diethyl etherate (> 47.5% BF3, Sigma Aldrich Inc.) was mixed with AIR material (approximately 5–10 mg) in a 1-mL screw-cap reaction vial. The vial cap was screwed on tightly and kept on a heating block at 100 °C for 4 h with gentle shaking. After cooling the vial in ice water for 5 min, 200 μL of product mixture solution was transferred into a new vial cap, and 100 μL of 1 M sodium hydrogen carbonate was added to adjust the pH to 7. Next, 130 μL of 1 M hydrochloric acid solution was used to adjust the pH to below 3. The resultant solution was extracted three times with diethyl ether (250 μL). The combined organic phase was washed with saturated sodium chloride and then evaporated after removing water contamination by adding anhydrous sodium sulfate. The residues were redissolved in approximately 250 μL of diethyl ether, and 10 μL of the solution was silylated by adding 8 μL of *N*, *O*-bis(trimethylsilyl)acetamide. The resulting solution was analyzed with a gas chromatograph−flame ionization detector (GC-2010 Plus, Shimadzu Inc.) equipped with a DB-5 capillary column (25 m × 0.25 mm, 0.25 μm film thickness, Agilent Technologies Inc.). The column oven temperature was maintained at 160 °C for the first 1 min, then increased at a rate of 10 °C min^−1^ to 300 °C. The split injector (1:10) was kept at 220 °C, and the FID was maintained at 300 °C. The flow speed of the carrier gas (nitrogen) was 30 cm/s. Conventional thioacidolysis monomers were quantified using the response factors provided by Yue et al. [33].

### Analysis of cell wall-bound phenolics

Levels of hydroxycinnamic acids linked to cell walls via ester or ether linkages were quantified by alkaline hydrolysis and subsequent analysis by GC. The AIR (50 mg) was treated with 4 mL of 4 N sodium hydroxide at 170 °C for 2 h. Tetracosane was added to the mixture as an internal standard. After removal by filtration of the residue, the solution was acidified with 1 N hydrochloric acid and saturated with sodium chloride. The solution was then extracted two times with diethylether and the ether-soluble fraction was recovered. This fraction was evaporated to dryness and the residue was dissolved in 250 μl of diethylether. The trimethylsilylated sample was analyzed by GC − FID (GC-2010 Plus, Shimadzu Inc.) equipped with DB-5 capillary column (25 m × 0.25mmID, 0.25 μm film thickness, Agilent Technologies Inc.). The column oven temperature was maintained at 120 °C for the first 2 min, then increased at 8 °C/min to 240 °C. The Split injector (1:10) was kept at 240 °C and the FID was kept at 300 °C. The flow speed of the carrier gas (nitrogen) was 30 cm/s. Although two hydroxybenzaldehydes (vanillin and syringaldehyde), two hydroxybenzoic acids (vanillic and syringic acids), and three hydroxycinnamic acids (*p*-coumaric, ferulic, and sinapic acids) were analyzed, differences among the tested samples were only detected in *p*-coumaric and ferulic acids (Table 1).

**Table 1:** Lignin content, lignin monomer composition, and level of cell-wall bound phenolics

Line	ASL (µg/mgAIR)	AIL (µg/mgAIR)	Total lignin (µg/mgAIR)	G unit (µmol/lignin)	S unit (µmol/lignin)	monomer yield (µmol/lignin)	S/G	PA	FA
Wild type	13 (0)	235 (20)	247 (19)	168 (27)	75 (12)	0.98 (0.12)	0.46 (0.03)	1	1
*PkC4Hpro::F5H*	21 (5)	186 (28)	207 (24)	39 (17)***	146 (42)**	0.89 (0.26)	11.13 (5.03)*	ND	ND
*PkC4Hpro::calB*	13 (1)	208 (15)	221 (15)	117 (32)**	58 (16)*	0.77 (0.21)*	0.51 (0.14)*	0.19	0.65
*PkC4Hpro::couA*	16 (1)***	211 (28)	227 (29)	68 (25)***	44 (15)**	0.49 (0.18)***	0.69 (0.22)***	4.0**	8.1**
*PkC4Hpro::F6H1*	13 (0)	231 (21)	244 (21)	91 (28)***	45 (14)**	0.55 (0.17)***	0.52 (0.15)*	ND	ND

ASL, acid soluble lignin expressed as µg/mgAIR

AIL, acid insoluble lignin expressed as µg/mgAIR

Total lignin, summed value of ASL and AIL

PA, cell-wall bound *p*-coumaric acid (expressed as relative level compared to wild type)

FA, cell-wall bound ferulic acid (expressed as relative level compared to wild type)

Each values indicate average. Numbers in parentheses are 95% confidential intervals

ND, not determined

*; *P*<0.05, **; *P*<0.01, ***; *P*<0.001

### Monosaccharide composition analysis

Monosaccharide composition was analyzed based on the UPLC-ABEE system as previously described [34]. AIR powder was hydrolyzed by two-step sulfuric acid hydrolysis, and the hydrolysate was neutralized with calcium carbonate. The neutralized supernatant was labeled with ethyl *p*-aminobenzoate (ABEE) reagent. Chromatographic separation and detection of labeled monosaccharides were performed using an ACQUITY UPLC H-Class system (Waters Inc.) equipped with an ACQUITY UPLC BEH C18 column (100 × 2.0 mm, 1.7 μm particle size, Waters Inc.) and fluorescence detector (ACQUITY UPLC FLR detector, Waters Inc.). The eluents used for the chromatographic separation were 200 mM potassium borate buffer (pH 8.9) and 100% acetonitrile.

### Sample pretreatment and enzymatic saccharification

A diluted alkali pretreatment was applied using a slight modification of the method of Santoro et al. [35]. Weighed AIR powder (1.5‒2.0 mg) was mixed with 870 μL of 100 mM NaOH solution and incubated for 2 h at 90 °C. After cooling to room temperature, the solution was neutralized with 20 μL of 30% (v/v) HCl, and saccharification was then carried out with 110 μL of neutralized solution containing cellulase [100 mM citrate (pH 4.5), 0.02% (w/v) sodium azide, 0.2 CPU cellulase (Celluclast^®^ 1.5 L, Merck Inc.), and 0.4 CBU cellobiase (Novozyme 188, Merck Inc.)]. After incubation for 24 h at 50 °C with shaking at 200 rpm, the amount of liberated glucose and xylose was determined with a Glucose CII test kit (Wako Inc.) and Xylose Assay kit (Megazyme Inc.), respectively. For non-pretreated samples, AIR powder was digested with 1,000 μL of cellulase solution [10 mM citrate (pH 4.5), 0.02% (w/v) sodium azide, 0.2 CPU cellulase (Celluclast^®^ 1.5 L, Merck Inc.), and 0.4 CBU cellobiase (Novozyme 188, Merck Inc.)] without any pretreatment.

### Statistical analysis

Using R software, the Shapiro–Wilk test (“shapiro.test” function) was used to determine if data were significantly likely to be normally distributed. If the data were assumed to be normally distributed, statistical analysis of growth parameters and biochemical data was performed using Welch’s *t* test with Bonferroni–Holm correction. However, if normal distribution was not significantly suggested (*p* < 0.05), the Brunner–Munzel test with Bonferroni–Holm correction was performed using the “brunner.munzel.test” function in the brunnermunzel package of R [36, 37].

## Results

### Promoter activity of *PkC4H* in *Arabidopsis*

To express enzymatic genes in *Arabidopsis* interfascicular and xylem fibers, the *C4H* gene promoter (*PkC4Hpro*) derived from hybrid aspen was selected in view of future application of the effective construct to woody plants. C4H is involved in lignin biosynthesis, hence *PkC4Hpro* is active in fiber cells and vessel precursor cells in hybrid aspen stems [21, 38]. To examine *PkC4Hpro* expression in *Arabidopsis*, the β-glucuronidase (GUS) gene fused with *PkC4Hpro* was stably introduced into the *Arabidopsis* genome, and GUS activity was monitored histochemically. GUS activity was evident in most tissues where secondary cell walls develop, including xylem and interfascicular fibers in inflorescence stems, root hypocotyls, siliques, and anther and leaf vasculature (Additional file 2: Fig. S1). This result suggested that the promoter activity was conserved as expected in *Arabidopsis* and thus appropriate for our purpose.

### Positive-control experiment using the *F5H* expression construct

**Fig. 2:**
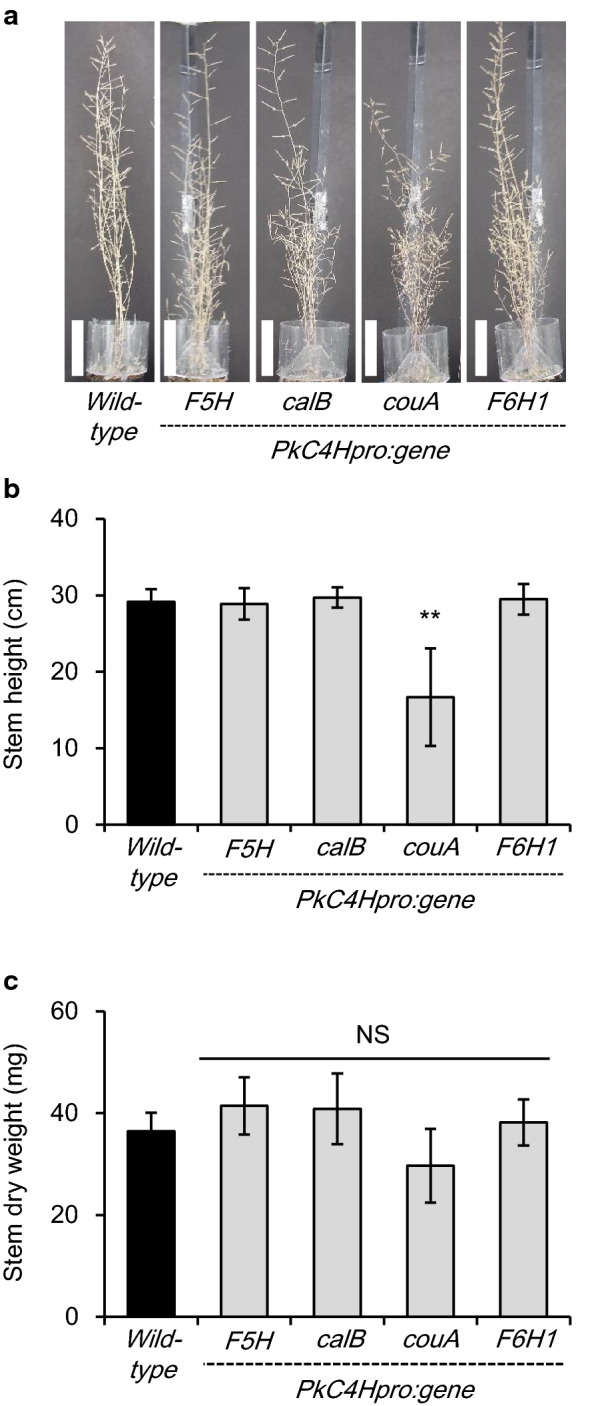
Lignin-modified transgenic plants exhibit no severe growth penalty. **a** Representative plants fully grown for 8 weeks and then dried for 4 weeks. Bars represent 5 cm. Stem height (**b**) and stem dry weight (**c**) of inflorescence stems of fully senesced transgenic lines (*n* = 9, wild-type; *n* = 8, *PkC4Hpro::F5H*; *n* = 9, *PkC4Hpro::calB*; *n* = 8, *PkC4Hpro::couA*; and *n* = 8, *PkC4Hpro::F6H1*) and the wild type. Error bars indicate the 95% confidence interval of the mean. Double asterisks indicate statistically significant differences (*P* < 0.01) of transgenic lines compared with the wild type according to Welch’s *t*-test or Brunner–Munzel test with Bonferroni–Holm correction

**Fig. 3:**
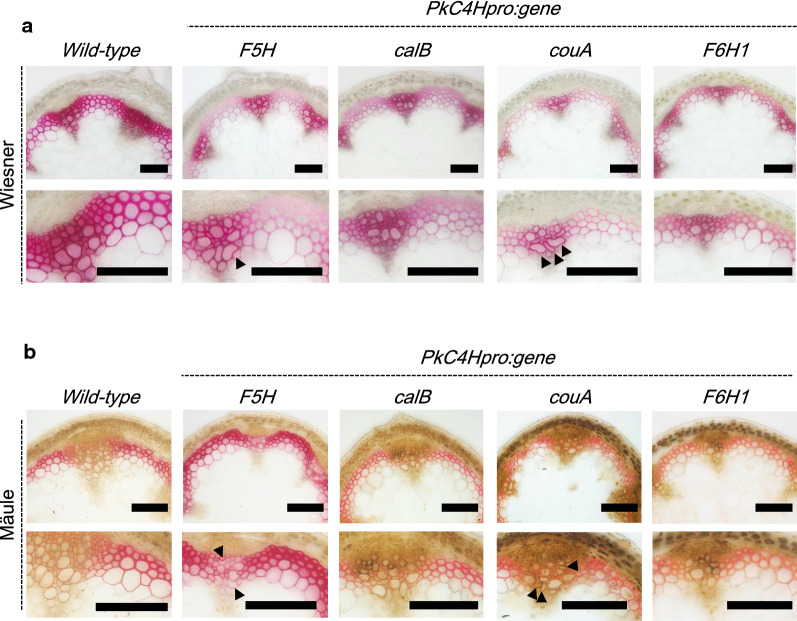
Wiesner (**a**) and Mäule (**b**) staining of hand-cut stem sections from transgenic plants harboring *F5H*, *calB*, *couA*, or *F6H1* transgenes. The sections were prepared from 8-week-old T_1_ transgenic plants generated by the transformation of single *Agrobacterium* strains harboring *PkC4Hpro::F5H, PkC4Hpro::calB, PkC4Hpro::couA,* or *PkC4Hpro::F6H1* constructs. The representative image for each line is presented. The lower panels are magnified images of corresponding upper panels. Scale bars = 100 μm

**Fig. 4:**
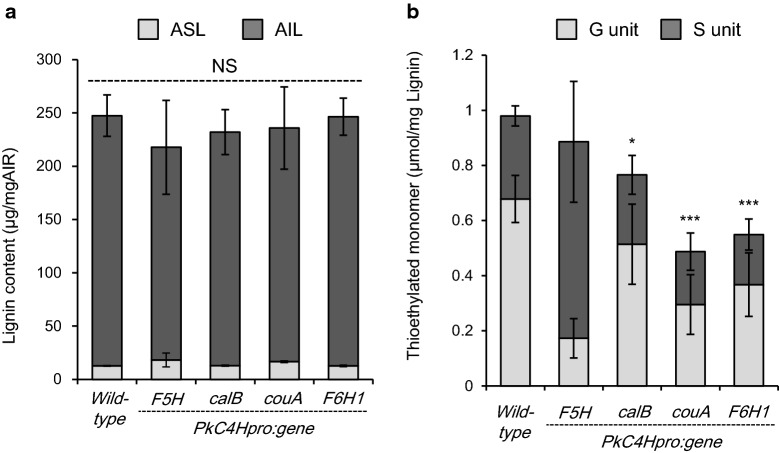
Lignin quantities of transgenic plants. Acid-insoluble lignin (AIL) and acid-soluble lignin (ASL) content (**a**), thioethylated monomer content (**b**) were measured in AIR prepared from inflorescence stems of wild-type and transgenic T_1_ plants harboring *PkC4Hpro::F5H, PkC4Hpro::calB, PkC4Hpro::couA,* or *PkC4Hpro::F6H1*. Error bars indicate 95% confidence intervals (*n* = 8 for the wild type, *PkC4Hpro::F5H*, *PkC4Hpro::couA, *and *PkC4Hpro::F6H1*; *n* = 9 for *PkC4Hpro::calB*). Single, double, and triple asterisks indicate statistically significant differences (* *P* < 0.05, ** *P* < 0.01, and *** *P* < 0.001, respectively) of transgenic lines compared with the wild type according to Welch’s *t*-test or Brunner–Munzel test with Bonferroni–Holm correction

To confirm that the prepared vector was suitable for our purpose, we used *F5H*/*CYP84A1* as a positive control. Overexpression of *F5H* under the control of the *Arabidopsis C4H* promoter has been found to increase the proportion of S units of lignin in an *Arabidopsis F5H*-deficient *fah1*-*2* mutant, wild-type tobacco, and poplar [8, 9]. This overexpression also reduces the lignin content of transgenic tobacco and poplar plants. In the case of our transgenic *Arabidopsis* expressing *F5H* under the control of *PkC4Hpro* (T_1_ generation), growth characteristics were similar to those of the wild type (Fig. 2); however, the color intensity of interfascicular fiber cells after lignin staining with Wiesner reagent (phloroglucinol–HCl solution; Fig. 3a) was weaker in multiple T_1_-independent lines than in the wild type, an observation also reported for *F5H*-overexpressing tobacco [8]. Furthermore, some vascular vessel elements were slightly distorted (triangle in Fig. 3a); this phenomenon, usually referred to as the “*irregular xylem* (*irx*)” phenotype, was not reported in the *fah1*-*2* mutant overexpressing *F5H* [9]. Vascular xylem cells and vessels of the F5H line were clearly stained in red by the Mäule reaction, while those of the wild type were stained in brown (Fig. 3b). As indicated by the results of py-GC/MS and thioacidolysis of AIR prepared from inflorescence stems, lignin in the F5H line was enriched with S units, as expected (Table 1, Fig. 4b, and Additional file 1: Table S4). From these observations, we concluded that the expected phenotypes were observed in the F5H line and that *PkC4Hpro* was thus appropriate for driving the expression of the tested genes in this study.

### Generation of transgenic plants and subsequent systematic screening by cross-sectional lignin staining

To test many enzyme-coding genes, we constructed 48 expression constructs encoding 50 different enzymes, including F5H, the positive control (Additional file 1: Table S1). All enzymes had the potential to act on metabolites in shikimate, general phenylpropanoid, flavonoid, or monolignol biosynthetic pathways (Additional file 1: Table S2). *Agrobacterium* strains harboring individual expression constructs were cultured independently, and two to eight cultures were mixed into a pool. *Arabidopsis* plants were transformed using the traditional floral-dip procedure and seven pools of *Agrobacterium* strains (see the column “Gene set” in Additional file 1: Table S3). After transformation and subsequent selection based on genomic PCR followed by sequencing of amplified DNA, we characterized 296 independent transgenic plants (T_1_ generation) harboring single or multiple transgenes (Additional file 1: Table S3). Nearly three-quarters of the transgenic plants carried a single transgene, whereas the rest carried two, three, or four transgenes (Additional file 1: Table S3). We failed to recover a transgenic plant carrying *Atu1417* in the present study.

The resulting transgenic plants were grown until inflorescence stems were fully elongated. Hand-sliced cross sections of primary inflorescence stems from all 296 transgenic plants were stained with Wiesner and Mäule reagents in 96-well plates (Additional file 2: Figs. S2 and S3). In this way, we found that some plants possessing at least one of the genes *calB*, *F6H1*, or *couA* in addition to the positive control, *F5H*, showed apparent abnormalities (Additional file 2: Figs. S2 and S3). In the analysis with the Mäule reaction, in particular, inflorescence stems of lines transgenic for *calB*, *couA*, and *F6H1* (CalB, CouA, and F6H1 lines, respectively) exhibited less staining in vascular xylem and interfascicular fibers (Additional file 2: Fig. S3).

### Confirmation of transgene effects in T_1_ generations derived from independent transformation procedures

To verify the effect of the transgenes observed above, we individually recreated T_1_ plants carrying each identified gene and compared them with wild type and the positive control line harboring the F5H sequence. As shown in Fig. 2a, b, the stem height of the couA line was shorter than that of the wild type even though other transgenic lines were almost the same as the wild type. The stem dry weight of the couA line was also slightly lower than the wild type, but not significantly different (Fig. 2c).

Although not extremely obvious (Additional file 2: Fig. S2 and S3), calB, couA, and F6H1 lines appeared to be more weakly stained by both Wiesner and Mäule reagents compared with the wild type, a situation particularly evident in interfascicular fiber cells of the newly generated individual T_1_ lines (Fig. 3). A typical *irx* phenotype was observed in vascular vessels of the couA line, where the red color generated in interfascicular fibers by Wiesner staining was apparently faint compared with that in calB and F6H1 lines. These data suggest that the expression of these genes leads to changes in lignin content and/or composition.

### Characterization of lignin and cell wall phenolics in transgenic lines

Lignin modification in the transgenic plant lines was confirmed by chemical analysis. Lignin content and the monomeric composition of lignin in senesced inflorescence stems were measured by micro-Klason, Py-GC/MS, and thioacidolysis procedures (Fig. 4, Tables 1 and 2, and Additional file 1: Table S4). The lignin content of all transgenic lines was not significantly different (Fig. 4a).

**Table 2:** Relative abundance of structural features within AIR-sample from wild-type and transgenic inflorescence stem on the basis of molar peak area identified by pyGC/MS

%	Wild type	*PkC4Hpro::F5H*	*PkC4Hpro::CalB*	*PkC4Hpro::couA*	*PkC4Hpro::F6H1*
H	12.06 (2.45)	4.08 (2.19)***	11.6 (1.16)	16 (1.93)*	11.86 (2.28)
G	60.6 (0.82)	17.89 (11.12)***	54.5 (3.3)***	49.41 (3.33)***	53.89 (4)***
S	27.34 (3)	78.03 (13.01)***	33.9 (2.74)**	34.56 (4.79)**	33.63 (5.18)*
Unsub	5.65 (1.26)	4.78 (1.13)	5.86 (0.71)	7.22 (0.71)	5.72 (0.95)
Methyl	3.23 (0.41)	2.14 (0.75)**	3.54 (0.42)	3.62 (0.42)	3.93 (0.55)*
Ethyl	0.2 (0.09)	0.12 (0.04)	0.22 (0.1)	0.44 (0.06)**	0.23 (0.11)
Vinyl	25.51 (4.23)	17.48 (3.82)**	26.87 (2.4)	35.43 (2.52)**	25.93 (3.36)
Cα-O	3.13 (0.25)	4.29 (0.42)***	3.48 (0.21)*	3.71 (0.24)**	3.72 (0.3)**
Cβ-O	1.46 (0.13)	1.02 (0.23)***	1.54 (0.09)	1.58 (0.07)	1.7 (0.24)*
Cγ-O	50.92 (7.12)	59.33 (8.48)	47.46 (4.36)	36.4 (3.63)**	46.66 (6.99)
Misc	9.9 (1.55)	10.83 (2.77)	11.02 (1.32)	11.59 (0.88)	11.48 (1.73)

Each values indicate average. Numbers in parentheses are 95% confidential intervals

*; P<0.05, **; P<0.01, ***; P<0.001

We next investigated lignin structure using thioacidolysis. Compared to wild type, a significant difference in the composition of two conventional monomers with G and S nuclei, indicators of relative amounts of β-*O*-4 structures in lignin, was observed in the calB, couA, and F6H1 lines (Fig. 4b and Table 1). The total levels of monomers released in these transgenic lines were also apparently reduced. These results indicate that expression of these genes leads to structural alteration of lignin without significant changes in lignin content. CalB can oxidize coniferaldehyde, sinapaldehyde, and benzaldehyde [39]. In addition, CouA is predicted to convert *p*-coumaroyl-CoA, caffeoyl-CoA, and feruloyl-CoA to their corresponding benzaldehydes [20]. It is thus presumed that levels of hydroxycinnamate and hydroxybenzaldehyde derivatives bound to cell walls are altered by the expression of these enzyme genes. However, contrary to expectations, no significant differences were observed in most of the phenolics released after alkaline hydrolysis of AIR, with the exception of *p*-coumaric and ferulic acids in the CouA line (Table 1).

**Fig. 5:**
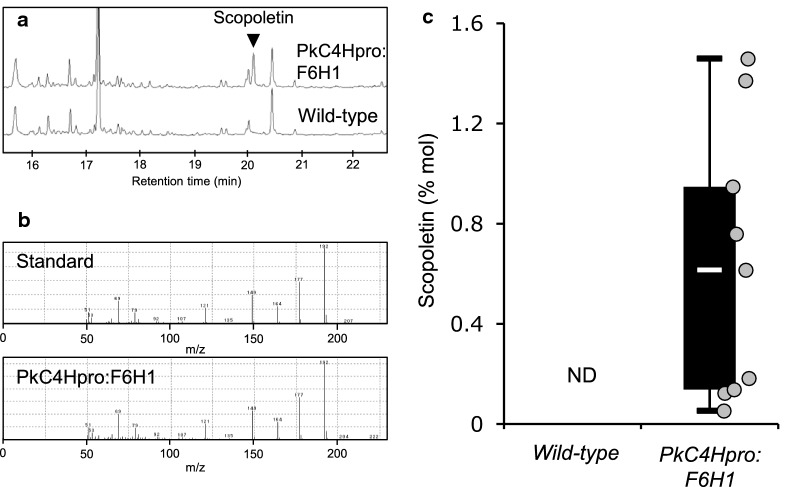
Detection of Scopoletin in F6H1 transgenic plants. Py-GC/MS was performed for inflorescence stem samples prepared from wild type and *PkC4Hpro::F6H1* plants fully grown for 8 weeks and completely dried for 4 weeks without watering. **a** Total ion chromatogram of pyrolyzed products from wild type and *PkC4Hpro::F6H1* lines. The triangle indicates the peak of scopoletin. **b** Mass spectra of pyrolyzed products of scopoletin. The upper and lower panels indicate mass spectra of scopoletin standard and pyrolyzed products, respectively, from the actual peak shown in (**a**). **c** Quantification of scopoletin content in AIR residue by Py-GC/MS. Boxplots represent the range of determined scopoletin content. Horizontal bars in the boxes indicate the median of the detected values. Upper and lower hinges of the boxes indicate 75% and 25% ranges of detected values, respectively. The upper and lower extreme bars of the boxplots indicate the maximum (upper) and the minimum (lower) of reported values, respectively. Each dot indicates raw scopoletin values in each F6H1 line

To further confirm the structural change of lignin, we analyzed the pyrolysis products derived from cell wall samples of each transgenic line by Py-GC/MS. As listed in Table 2, the relative distribution of S units in all transgenic lines was slightly increased while that of G units was decreased. The data are consistent with the results of thioacidolysis, which indicated higher S/G ratio in the transgenic lines compared to the wild type. In CouA lines, vinyl-substituted products are noticeably more abundant than in the wild type. Van Ervan et al. [31] reported that vinylic pyrolysis products such as 4-vinylphenol and 4-vinyl guaiacol are derived from interunit linkages of lignin and wall-bound phenolics such as *p*-coumarate and ferulate. This data therefore suggests that these hydroxycinnamic acids bound to cell wall are more abundant in CouA line. Increase of these acids was actually observed by the alkaline hydrolysis followed by GC analysis (Table 1). Another marked difference was a decrease of Cγ-O pyrolyzed products in CouA lines. According to Van Erven et al. [40], these products are primarily derived from lignin interunit linkages with a three-carbon side-chain. Most of the Cγ-O products should be derived from β-*O*-4 structures in lignin since our pyrolysis conditions gave no secondary reaction to the initial three-carbon side-chain connected with β-*O*-4 linkage. This is consistent with the reduction of total monomer yield in CouA lines. In F6H1 lines, we detected scopoletin in the AIR sample using Py-GC/MS (Fig. 5). F6H1 is a key enzyme for scopoletin biosynthesis, primarily through feruloyl-CoA 6′-hydroxylation in plant roots [41]. Given this, our strategy using the F6H1 enzyme worked for the production of scopoletin bound to the cell wall, more specifically, possibly to the lignin in xylem tissues.

### Monosaccharide composition and saccharification efficiency

**Fig. 6:**
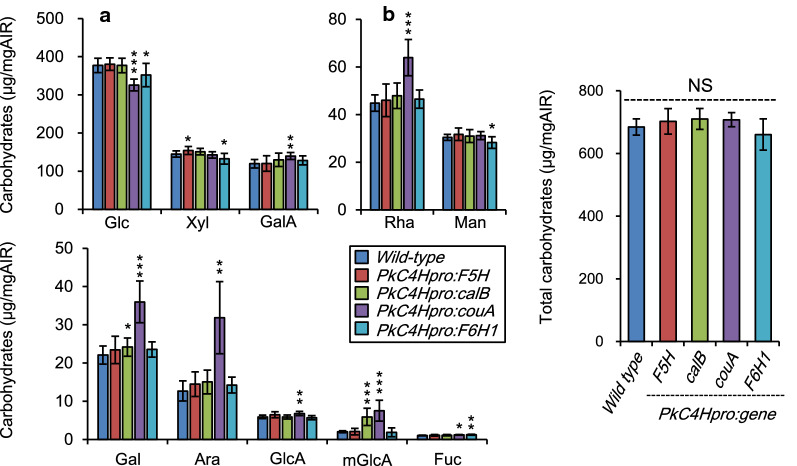
Monosaccharide composition of cell walls of transgenic plants. Each individual monosaccharide (**a**) the total amounts of 10 monosaccharides (**b**) in cell walls of fully grown for 8 weeks and completely dried for 4 weeks plants without watering and are shown. Glc, Xyl, GalA, Rha, Man, Gal, Ara, mGlcA, GlcA, and Fuc represent glucose, xylose, galacturonic acid, rhamnose, mannose, galactose, arabinose, 4-methyl-glucuronic acid, glucuronic acid, and fucose, respectively. Error bars indicate 95% confidence intervals (*n* = 8 for the wild type; *n* = 16 for *PkC4Hpro::F5H* and *PkC4Hpro::calB*; *n* = 11 for *PkC4Hpro::couA; n* = 14 for *PkC4Hpro::F6H1*). Asterisks indicate statistically significant differences (* *P* < 0.05, ** *P* < 0.01, and *** *P* < 0.001, respectively) of transgenic lines compared with the wild type according to Welch’s *t*-test or Brunner–Munzel test with Bonferroni–Holm correction

We next analyzed the monosaccharide composition of AIR prepared from each transgenic line. We found slight changes in monosaccharide composition in CouA and F6H1 lines compared with the wild type, namely, glucose was decreased and 4-*O*-methyl-glucuronic acid was increased (Fig. 6a and Additional file 1: Table S5). In the CouA line, galactose, arabinose, and rhamnose were increased as well (Fig. 6a). However, total sugars per unit of AIR were not significantly changed in any of these lines from wild type (Fig. 6b and Additional file 1: Table S5). These results indicate that monosaccharide composition was affected by the modification of lignin in the transgenic lines.

**Fig. 7:**
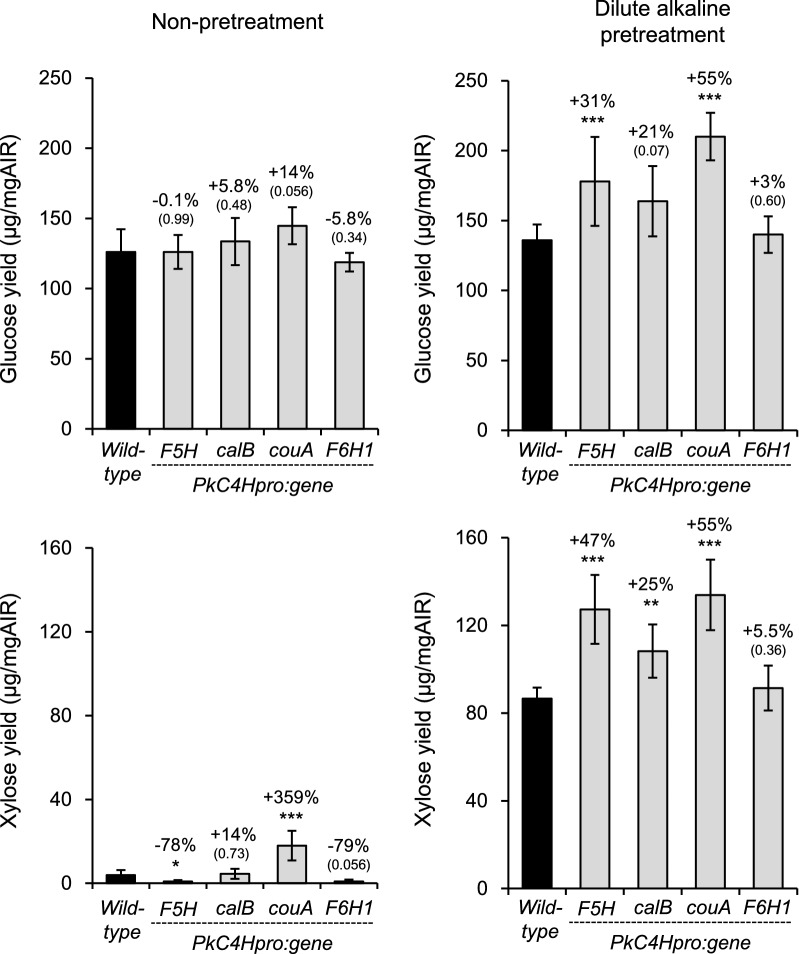
Lignin engineered transgenic plants improve sugar yield by mild alkali pretreatment. **a**, **b** Glucose yield by enzymatic saccharification of dried inflorescence stems from wild type and transgenic plants grown for 8 weeks and completely dried for 4 weeks without watering (*PkC4Hpro::F5H*, *PkC4Hpro::calB*, *PkC4Hpro::couA*, and *PkC4Hpro::F6H1*) without pretreatment (**a**) or with dilute alkaline pretreatment (**b**). **c**, **d** Released xylose by the enzymatic saccharification from the cell wall residue of wild-type and transgenic plants without pretreatment (**c**) or with dilute alkaline pretreatment. Error bars indicate 95% confidential intervals (**a**, **c***n* = 8, wild-type; *n* = 11, *PkC4Hpro::F5H*; *n* = 14, *PkC4Hpro::calB*; *n* = 10, *PkC4Hpro::couA*; *n* = 8, *PkC4Hpro::F6H1*. **b**, **d**; *n* = 8, wild-type; *n* = 13, *PkC4Hpro::F5H*; *n* = 16, *PkC4Hpro::calB*; *n* = 11, *PkC4Hpro::couA*; *n* = 11, *PkC4Hpro::F6H1*). Scores above each bars were fold-change from wild-type (upper) and *P* values (lower, * *P* < 0.05, ** *P* < 0.01, *** *P* < 0.001, respectively) according to Welch’s t-test or Brunner–Munzel test with Bonferroni–Holm correction compared with wild type

To further evaluate the impact of lignin modification on cell wall characteristics of the transgenic lines, we measured levels of glucose and xylose released after enzymatic saccharification to determine the saccharification efficiency of AIR with or without alkaline pretreatment (Fig. 7 and Additional file 1: Table S6). Although not improved significantly without pretreatment, glucose release was increased after pretreatment in CalB, CouA, and F5H (positive control) lines, by 21%, 55%, and 31%, respectively, compared with the wild type. Although the efficiency varied, xylose release was also enhanced significantly by pretreatment in the same transgenic lines. Without pretreatment, improved release of xylose compared with the wild type was observed only in the CouA line. While lignin composition was changed just as in other transgenic lines, no improved sugar release was observed in the F6H1 line.

## Discussion

Lignin manipulation is one of the major techniques for reducing lignocellulose recalcitrance. Manipulation can be achieved by suppressing endogenous genes for lignin biosynthesis [7, 42, 43], and by the expression of heterologous genes to prompt biosynthesis of particular phenolics that can act as alternative lignin monomers [44–46]. To our knowledge, this is the first attempt to simultaneously screen a large variety of enzymes for the manipulation of lignin. The screening results were supported by the recreated individual T_1_ lines. Changes in lignin composition (Fig. 4, Table 1 and Additional file 1: Table S4) and saccharification efficiency (Fig. 7 and Additional file 1: Table S6) were clearly observed in these T_1_ lines. Our results indicate that systematic screening via a histochemical staining assay in *Arabidopsis* is adequate to identify lignin modifier genes.

calB encodes coniferaldehyde dehydrogenase from *Pseudomonas* sp. HR199, which catalyzes the NAD^+^-dependent oxidation of coniferaldehyde and sinapaldehyde to their corresponding hydroxycinnamic acids (Fig. 1 and Additional file 1: Tables S1 and S2) [39, 47]. calB expression was expected to lead to a reduction in conifer- and sinapaldehyde accumulation and an increase in their corresponding acids. This hypothesis was partially supported by the reduced intensity of red color in interfascicular fibers, as detected by Wiesner staining. However, contrary to expectations, levels of cell wall-bound *p*-coumaric and ferulic acids seemed to be lower compared to the wild type, while sinapic acid levels were lower than the detection limit in both the calB and wild-type lines (Table 1 and Additional file 1: Table S4). One of the reasons for this might be a significantly higher *K*_*m*_ value of calB for coniferaldehyde (334 μM) [39] compared to those of *Arabidopsis* CAD-4 (65 μM) and CAD-5 (35 μM) [48], which have major role in the reduction of coniferaldehyde for monolignol biosynthesis. However, monomeric composition of lignin was apparently altered, and saccharification efficiency was significantly improved in the calB line, though the mechanism is unclear. Future studies should address the role of calB in improved lignocellulose recalcitrance.

The *couA* gene of *Rhodopseudomonas palustris* encodes a couA polypeptide that catalyzes side-chain cleavage of hydroxycinnamoyl-CoAs to generate acetyl-CoA and corresponding HBAlds [20], similarly to *Pseudomonas fluorescens* HCHL [19]. In one study, expression of *hchl* under the control of the xylem-specific *cellulose synthase A4* promoter in *Arabidopsis* induced the *irx* phenotype in one of five independent transgenic lines. However, Wiesner and Mäule staining patterns in stem sections of the HCHL lines remained similar to those in the wild type [17]. In our study, by contrast, the couA line exhibited the typical *irx* phenotype with collapsed vessels (Fig. 3), along with faint coloration in Wiesner staining, especially in interfascicular fibers. Reduction of thioacidolysis monomer yield without decrease in total lignin content was observed in the HCHL line as well as couA line while the reduction in couA line (Table 1) was apparently larger than that in the HCHL lines [17]. This might be due to differences in catalytic properties of the enzymes, transcriptional activity of the employed promoters, generation of the analyzed transgenic plants (T_2_ in HCHL lines vs. T_1_ in couA lines), or a combination of these. Lignin modification often causes change in monosaccharide composition [49]. The impact of gene expression on the monomeric composition of cell wall polysaccharides was observed in the couA line (Fig. 6 and Additional file 1: Table S6). Interestingly, monosaccharide composition of couA lines in our study is changed from wild type; especially Rha, Gal, and Ara, major components of pectin, were clearly increased, suggesting a less developed secondary cell wall in the transgenic plants. In any case, the preferential expression of couA achieved lignin manipulation and subsequent improved sugar release from the transgenic plants without biomass yield penalty.

Similar to couA, F6H1 recognizes feruloyl-CoA, a key intermediate in monolignol biosynthesis, as a substrate. This enzyme is derived from *A. thaliana* and converts feruloyl-CoA to 6′-hydroxyferuloyl-CoA and 2-oxoglutaric acid (Additional file 1: Table S2) [50, 51]. The resultant 6′-hydroxyferuloyl-CoA is then subjected to non-enzymatic lactonization to generate 6-methoxyumbelliferone (scopoletin) [50]. Scopoletin is well known as a phytoalexin. The genes for its biosynthesis are mainly expressed in roots, where lignin content is limited, but scarcely expressed in stems [40]. While scopoletin is usually accumulated in the vacuole in its glycosylated form, in our study scopoletin was detected from alcohol-insoluble residue regarded as cell wall fraction. This suggests that scopoletin can be transported to the cell wall and then incorporated into lignin, cell wall polysaccharides, or both. A new lignin chain is initiated from its hydroxy residue and terminated at the same site. Even though it is still unclear whether scopoletin is incorporated into the transgenic plant lignin, it has the capacity to act as a lignin initiator and terminator. It is considered that these potential roles of scopoletin could improve enzyme saccharification efficiency of the cell wall as reported previously for both unusual initiator and terminator [17, 52]. Further characterization of lignin structure including studying the distribution of molecular weight will clarify the effect of scopoletin on lignin polymerization.

## Conclusions

Efficient mining of candidate genes that manipulate lignin biosynthesis can be accomplished by in planta screening of numerous introduced genes in transgenic *Arabidopsis* based on staining of stem tissues using two different lignin-staining procedures. In-depth characterization of lignin structure using chemical methods accompanied by investigation of transgene expression and metabolites would provide valuable information for the reduction of biomass recalcitrance by using the identified genes. It should be noted that there are other efficient methods of screening like Fourier transform infrared spectrometer (FTIR) analysis and near infrared spectroscopy analysis (NIRS) and the application of our samples to them would uncover more genes which could change lignin.

## Supplementary information

**Additional file 1: Table S1.** List of genes used for lignin modification. **Table S2.** Summary of properties of enzymes involved in the conversion of metabolites in shikimate, general phenylpropanoid, flavonoid, or monolignol biosynthetic pathways. Reaction scheme, coenzyme requirement, enzyme activity (kinetic parameters, specific activity, or substrate range), and reference(s) are shown. **Table S3.** List of gene sets and number of plants harboring each gene. The number of transgenic plants having a single transgene or multiple transgenes is indicated in the third column. Efficiency refers to the relative proportion of plants harboring each gene among all plants generated by the same mixture of *Agrobacterium* strains. **Table S4.** Relative abundance of pyrolysis products within AIR-sample from wild-type and transgenic inflorescence stem. **Table S5.** Carbohydrates composition in transgenic lines supporting Fig. 6. **Table S6.** Sugar yield by enzymatic saccharification supporting Fig. 7.

**Additional file 2: Fig. S1.** Histochemical GUS staining analysis. ß-glucuronidase (GUS) activity was monitored in transgenic Arabidopsis plants harboring a gene for GUS fused with *PkC4Hpro* (A, C, E, G, I, K, M, O, Q, S, U, and V). Lignin autofluorescence was also observed in each sample (B, D, F, H, J, L, N, P, R, and T). C, D, G, H, K, L, O, P, S, and T are the magnified views of A, B, E, F, I, J, M, N, Q, and R, respectively. **Fig. S2.** Wiesner staining of hand-cut sections. Samples were prepared from primary inflorescence stems of T_1_ plants harboring the single gene shown in each panel. Staining procedures were performed in 96-well plates. The diameter of each well was 7 mm. Bars indicate 150 µm. **Fig. S3.** Mäule staining of hand-cut sections. Samples were prepared from primary inflorescence stems of T_1_ plants harboring the single gene shown in each panel. Staining procedures were performed in 96-well plates. The diameter of each well was 7 mm. Bars indicate 150 µm.

## Data Availability

All data generated or analyzed during this study are included in this published article and its Additional files 1, 2.

## Abbreviations

calB: Coniferaldehyde dehydrogenase
F6H1: Feruloyl-CoA 6′-hydroxylase
couA: Hydroxycinnamoyl-CoA hydratase/lyase
F5H: Ferulate 5-hydroxylase
C4H: Cinnamate 4-hydroxylase
CF: Coniferyl ferulate
HCHL: Hydroxycinnamoyl-CoA hydratase/lyase
HBAld: Hydroxybenzaldehyde
AIR: Alcohol insoluble residue
ABEE: *p*-Aminobenzoate
IRX: Irregular xylem
FTIR: Fourier transform infrared spectrometer
NIRS: Near-infrared spectroscopy analysis

## References

[1] Boerjan W, Ralph J, Baucher M (2003). Lignin biosynthesis. Annu Rev Plant Biol.

[2] Moura JC, Bonine CA, de Viana OFJ, Dornelas MC, Mazzafera P (2010). Abiotic and biotic stresses and changes in the lignin content and composition in plants. J Integr Plant Biol.

[3] Kitin P, Voelker SL, Meinzer FC, Beeckman H, Strauss SH, Lachenbruch B (2010). Tyloses and phenolic deposits in xylem vessels impede water transport in low-lignin transgenic poplars: a study by cryo-fluorescence mcroscopy. Plant Physiol.

[4] Vanholme R, Demedts B, Morreel K, Ralph J, Boerjan W (2010). Lignin biosynthesis and structure. Plant Physiol.

[5] Ralph J, Brunow G, Harris PJ, Dixon RA, Schatz PF, Boerjan W, Daayf F, Lattanzio V (2008). Lignification are lignins biosynthesized via simple combinatorial chemistry or via proteinaceous control and template replication?. Recent advances in polyphenol research.

[6] Himmel ME, Ding SY, Johnson DK, Adney WS, Nimlos MR, Brady JW, Foust TD (2007). Biomass recalcitrance: engineering plants and enzymes for biofuels production. Science.

[7] Van Acker R, Vanholme R, Storme V, Mortimer JC, Dupree P, Boerjan W (2013). Lignin biosynthesis perturbations affect secondary cell wall composition and saccharification yield in *Arabidopsis thaliana*. Biotechnol Biofuels.

[8] Franke R, McMichael CM, Meyer K, Shirley AM, Cusumano JC, Chapple C (2000). Modified lignin in tobacco and poplar plants over-expressing the *Arabidopsis* gene encoding ferulate 5-hydroxylase. Plant J..

[9] Meyer K, Shirley AM, Cusumano JC, Bell-Lelong DA, Chapple C (1998). Lignin monomer composition is determined by the expression of a cytochrome P450-dependent monooxygenase in *Arabidopsis*. Proc Natl Acad Sci USA.

[10] Stewart JJ, Akiyama T, Chapple C, Ralph J, Mansfield SD (2009). The effects on lignin structure of overexpression of ferulate 5-hydroxylase in hybrid poplar. Plant Physiol.

[11] Li X, Ximenes E, Kim Y, Slininger M, Meilan R, Ladisch M, Chapple C (2010). Lignin monomer composition affects *Arabidopsis* cell-wall degradability after liquid hot water pretreatment. Biotechnol Biofuels.

[12] Huntley SK, Ellis D, Gilbert M, Chapple C, Mansfield SD (2003). Significant increases in pulping efficiency in C4H-F5H-transformed poplars: improved chemical savings and reduced environmental toxins. J Agric Food Chem.

[13] Wilkerson CG, Mansfield SD, Lu F, Withers S, Park JY, Karlen SD, Gonzales-Vigil E, Padmakshan D, Unda F, Rencoret J (2014). Monolignol ferulate transferase introduces chemically labile linkages into the lignin backbone. Science.

[14] Kim KH, Dutta T, Ralph J, Mansfield SD, Simmons BA, Singh S (2017). Impact of lignin polymer backbone esters on ionic liquid pretreatment of poplar. Biotechnol Biofuels.

[15] Zhou S, Runge T, Karlen SD, Ralph J, Gonzales-Vigil E, Mansfield SD (2017). Chemical pulping advantages of Zip-lignin hybrid poplar. Chemsuschem.

[16] Oyarce P, De Meester B, Fonseca F, de Vries L, Goeminne G, Pallidis A, De Rycke R, Tsuji Y, Li Y, Van den Bosch S (2019). Introducing curcumin biosynthesis in *Arabidopsis* enhances lignocellulosic biomass processing. Nat Plants..

[17] Eudes A, George A, Mukerjee P, Kim JS, Pollet B, Benke PI, Yang F, Mitra P, Sun L, Cetinkol OP (2012). Biosynthesis and incorporation of side-chain-truncated lignin monomers to reduce lignin polymerization and enhance saccharification. Plant Biotechnol J.

[18] Mahon EL, Mansfield SD (2019). Tailor-made trees: engineering lignin for ease of processing and tomorrow’s bioeconomy. Curr Opin Biotechnol.

[19] Mitra A, Kitamura Y, Gasson MJ, Narbad A, Parr AJ, Payne J, Rhodes MJ, Sewter C, Walton NJ (1999). 4-hydroxycinnamoyl-CoA hydratase/lyase (HCHL)-an enzyme of phenylpropanoid chain cleavage from *Pseudomonas*. Arch Biochem Biophys.

[20] Hirakawa H, Schaefer AL, Greenberg EP, Harwood CS (2012). Anaerobic *p*-coumarate degradation by *Rhodopseudomonas palustris* and identification of CouR, a MarR repressor protein that binds *p*-coumaroyl coenzyme A. J Bacteriol.

[21] Kawai S, Mori A, Shiokawa T, Kajita S, Katayama Y, Morohoshi N (1996). Isolation and analysis of cinnamic acid 4-hydroxylase homologous genes from a hybrid aspen, *Populus kitakamiensis*. Biosci Biotechnol Biochem..

[22] Clough SJ, Bent AF (1998). Floral dip: a simplified method for Agrobacterium-mediated transformation of *Arabidopsis thaliana*. Plant J..

[23] Karp PD, Riley M, Paley SM, Pellegrini-Toole A (2002). The MetaCyc database. Nucleic Acids Res.

[24] Kanehisa M, Goto S (2000). KEGG: kyoto encyclopedia of genes and genomes. Nucleic Acids Res.

[25] Leinonen R, Diez F, Binns D, Fleischmann W, Lopez R, Apweiler R (2004). UniProt archive. Bioinformatics.

[26] Schomburg I, Chang A, Hofmann O, Ebeling C, Ehrentreich F, Schomburg D (2002). BRENDA: a resource for enzyme data and metabolic information. Trends Biochem Sci.

[27] Nagaya S, Kawamura K, Shinmyo A, Kato K (2010). The HSP terminator of *Arabidopsis thaliana* increases gene expression in plant cells. Plant Cell Physiol.

[28] Nakagawa T, Suzuki T, Murata S, Nakamura S, Hino T, Maeo K, Tabata R, Kawai T, Tanaka K, Niwa Y (2007). Improved gateway binary vectors: high-performance vectors for creation of fusion constructs in transgenic analysis of plants. Biosci Biotechnol Biochem.

[29] Sakamoto S, Takata N, Oshima Y, Yoshida K, Taniguchi T, Mitsuda N (2016). Wood reinforcement of poplar by rice NAC transcription factor. Sci Rep..

[30] Sakamoto S, Mitsuda N (2015). Reconstitution of a secondary cell wall in a secondary cell wall-deficient *Arabidopsis* mutant. Plant Cell Physiol.

[31] Van Erven G, de Visser R, Merkx DW, Strolenberg W, de Gijsel P, Gruppen H, Kabel MA (2017). Quantification of lignin and its structural features in plant biomass using 13C lignin as internal standard for pyrolysis-GC-SIM-MS. Anal Chem.

[32] Yamamura M, Hattori T, Suzuki S, Shibata D, Umezawa T (2012). Microscale thioacidolysis method for the rapid analysis of β-O-4 substructures in lignin. Plant Biotechnol..

[33] Yue F, Lu F, Sun RC, Ralph J (2012). Syntheses of lignin-derived thioacidolysis monomers and their uses as quantitation standards. J Agric Food Chem.

[34] Sakamoto S, Yoshida K, Sugihara S, Mitsuda N (2015). Development of a new high-throughput method to determine the composition of ten monosaccharides including 4-*O*-methyl glucuronic acid from plant cell walls using ultra-performance liquid chromatography. Plant Biotechnol..

[35] Santoro N, Cantu SL, Tornqvist CE, Falbel TG, Bolivar JL, Patterson SE, Pauly M, Walton JD (2010). A high-throughput platform for screening milligram quantities of plant biomass for lignocellulose digestibility. Bioenerg Res..

[36] Brunner E (2000). Munzel U: the nonparametric Behrens-Fisher problem: Asymptotic theory and a small-sample approximation. Biom J.

[37] Neubert K, Brunner E (2007). A studentized permutation test for the non-parametric Behrens-Fisher problem. Comput Stat Data Anal.

[38] Ro DK, Mah N, Ellis BE, Douglas CJ (2001). Functional characterization and subcellular localization of poplar (*Populus trichocarpa *× *Populus deltoides*) cinnamate 4-hydroxylase. Plant Physiol.

[39] Achterholt S, Priefert H, Steinbuchel A (1998). Purification and characterization of the coniferyl aldehyde dehydrogenase from *Pseudomonas* sp. strain HR199 and molecular characterization of the gene. J Bacteriol..

[40] Van Erven G, Nayan N, Sonnenberg ASM, Hendriks WH, Cone JW, Kabel MA (2018). Mechanistic insight in the selective delignification of wheat straw by three white-rot fungal species through quantitative C-IS py-GC-MS and whole cell wall HSQC NMR. Biotechnol Biofuels.

[41] Schmid NB, Giehl RF, Doll S, Mock HP, Strehmel N, Scheel D, Kong X, Hider RC, von Wiren N (2014). Feruloyl-CoA 6′-Hydroxylase1-dependent coumarins mediate iron acquisition from alkaline substrates in *Arabidopsis*. Plant Physiol.

[42] Van Acker R, Leple JC, Aerts D, Storme V, Goeminne G, Ivens B, Legee F, Lapierre C, Piens K, Van Montagu MC (2014). Improved saccharification and ethanol yield from field-grown transgenic poplar deficient in cinnamoyl-CoA reductase. Proc Natl Acad Sci USA.

[43] O’Connell A, Holt K, Piquemal J, Grima-Pettenati J, Boudet A, Pollet B, Lapierre C, Petit-Conil M, Schuch W, Halpin C (2002). Improved paper pulp from plants with suppressed cinnamoyl-CoA reductase or cinnamyl alcohol dehydrogenase. Transgenic Res.

[44] Grabber JH, Schatz PF, Kim H, Lu F, Ralph J (2010). Identifying new lignin bioengineering targets: 1. Monolignol-substitute impacts on lignin formation and cell wall fermentability. BMC Plant Biol..

[45] Vanholme R, Morreel K, Darrah C, Oyarce P, Grabber JH, Ralph J, Boerjan W (2012). Metabolic engineering of novel lignin in biomass crops. New Phytol.

[46] Mottiar Y, Vanholme R, Boerjan W, Ralph J, Mansfield SD (2016). Designer lignins: harnessing the plasticity of lignification. Curr Opin Biotechnol.

[47] Overhage J, Steinbüchel A, Priefert H (2002). Biotransformation of eugenol to ferulic acid by a recombinant strain of *Ralstonia eutropha* H16. Appl Environ Microbiol.

[48] Kim SJ, Kim MR, Bedgar DL, Moinuddin SGA, Cardenas CL, Davin LB, Kang CH, Lewis NG (2004). Functional reclassification of the putative cinnamyl alcohol dehydrogenase multigene family in *Arabidopsis*. Proc Natl Acad Sci.

[49] Van Acker R, Vanholme R, Storme V, Mortimer JC, Dupree P, Boerjan W (2013). Lignin biosynthesis perturbations affect secondary cell wall composition and saccharification yield in *Arabidopsis thaliana*. Biotechnol Biofuel..

[50] Kai K, Mizutani M, Kawamura N, Yamamoto R, Tamai M, Yamaguchi H, Sakata K, Shimizu B (2008). Scopoletin is biosynthesized via *ortho*-hydroxylation of feruloyl CoA by a 2-oxoglutarate-dependent dioxygenase in *Arabidopsis thaliana*. Plant J..

[51] Sun X, Zhou D, Kandavelu P, Zhang H, Yuan Q, Wang BC, Rose J, Yan Y (2015). Structural insights into substrate specificity of feruloyl-CoA 6′-hydroxylase from *Arabidopsis thaliana*. Sci Rep..

[52] Zhang K, Bhuiya MW, Pazo JR, Miao Y, Kim H, Ralph J, Liu CJ (2012). An engineered monolignol 4-*O*-methyltransferase depresses lignin biosynthesis and confers novel metabolic capability in *Arabidopsis*. Plant Cell..

