# Supplementary material for: Identification of enzymatic genes with the potential to reduce biomass recalcitrance through lignin manipulation in Arabidopsis
Source: Biotechnol Biofuels. 2020 May 29;13:97. doi: 10.1186/s13068-020-01736-6 (PMC7260809; doi:10.1186/s13068-020-01736-6)
Supplement: Supplementary file 1 — Additional file 1: Table S1. List of genes used for lignin modification. Table S2. Summary of properties of enzymes involved in the conversion of metabolites in shikimate, general phenylpropanoid, flavonoid, or monolignol biosynthetic pathways. Reaction scheme, coenzyme requirement, enzyme activity (kinetic parameters, specific activity, or substrate range), and reference(s) are shown. Table S3. List of gene sets and number of plants harboring each gene. The number of transgenic plants having a single transgene or multiple transgenes is indicated in the third column. Efficiency refers to the relative proportion of plants harboring each gene among all plants generated by the same mixture of Agrobacterium strains. Table S4. Relative abundance of pyrolysis products within AIR-sample from wild-type and transgenic inflorescence stem. Table S5. Carbohydrates composition in transgenic lines supporting Fig. 6. Table S6. Sugar yield by enzymatic saccharification supporting Fig. 7. [file 13068_2020_1736_MOESM1_ESM.pdf]

Table S1. List of genes used for lignin modification.

Gene	Function	Organism	Kingdom	Locus tag/ Gene symbol	Accession	Reference
F5H	ferulic acid 5-hydroxylase	*Arabidopsis thaliana*	Viridiplantae	AT4G36220	AEE86636	[1]
3AT2	coumaroyl-CoA:anthocyanidin 3-*O*-glucoside-6''-*O*-coumaroyltransferase	*Arabidopsis thaliana*	Viridiplantae	AT1G03495	AAF86513	[2]
ACT	agmatine coumaroyltransferase	*Hordeum vulgare*	Viridiplantae	–	BAF97626	[3]
AT208	anthocyanidin 3-*O*-glucoside 6'-*O*-acyltransferase	*Perilla frutescens*	Viridiplantae	–	BAA93475	[4]
Atu1417	enoyl-CoA hydratase	*Agrobacterium fabrum* C58	Bacteria	Atu1417	AAK87210	[5]
Balat_0669	chlorogenic acid esterase	*Bifidobacterium animalis* DSM 10140	Bacteria	Balat_0669	ACS47609	[6]
BrRCD	caffeic acid 4,5-dioxygenase	*Bradyrhizobium* sp. ORS278	Bacteria	BRADO_RS26060	WP_012029184	[7]
C2H	*p*-coumaroyl 2'-hydroxylase	*Ipomoea batatas*	Viridiplantae	–	BAL22347	[8]
CA9OMT	coniferyl alcohol 9-*O*-methyltransferase	*Linum nodiflorum*	Viridiplantae	–	ABX71750	[9]
CADH	coniferyl alcohol dehydrogenase	*Streptomyces* sp. NL15-2K	Bacteria	–	BAN09098	[10]
CALipB	lipase B	*Moesziomyces antarcticus*	Fungi	–	CAA83122	[11]
calA	coniferyl alcohol dehydrogenase	*Pseudomonas* sp. HR199	Bacteria	–	CAB69495	[12]
calB	coniferyl aldehyde dehydrogenase	*Pseudomonas* sp. HR199	Bacteria	–	CAA06926	[13]
CASHT	hydroxycinnamoyl-CoA: serotonin *N*-(hydroxycinnamoyl)transferase	*Capsicum annuum*	Viridiplantae	LOC107839132	AAK15312	[14]
CCMT1	cinnamic acid/*p*-coumaric acid carboxyl methyltransferase	*Ocimum basilicum*	Viridiplantae	–	ABG75942	[15]
CGT	chlorogenic acid: glucaric acid caffeoyltransferase	*Solanum lycopersicum*	Viridiplantae	LOC101265588	CBV37053	[16]
CHS5	chalcone synthase	*Glycine max*	Viridiplantae	GLYMA_08G109200	AAB01004	[17]
couA	*hydroxycinnamoyl-CoA hydratase-lyase*	*Rhodopseudomonas palustris* CGA009	Bacteria	RPA1786	CAE27227	[18]
CURS3	curcumin synthase	*Curcuma longa*	Viridiplantae	–	BAH85781	[19]
CYP84A4	*p*-coumaraldehyde 3-hydroxylase	*Arabidopsis thaliana*	Viridiplantae	At5g04330	AED90728	[20]
CYP98A22	*p*-coumaroyl 2'-hydroxylase	*Ruta graveolens*	Viridiplantae	CYP98A22	AEG19446	[21]
F6H1	feruloyl-CoA 6'-hydroxylase	*Arabidopsis thaliana*	Viridiplantae	AT3G13610	AEE75382	[22]
FDC1	phenacrylic acid decarboxylase	*Saccharomyces cerevisiae* S288C	Fungi	YDR539W	DAA12368	[23]
HCALDH	hydroxycinnamaldehyde dehydrogenase	*Arabidopsis thaliana*	Viridiplantae	AT3G24503	AEE76907	[24]
HCBT	anthranilic acid *N*-hydroxycinnamoyl /benzoyltransferase	*Dianthus caryophyllus*	Viridiplantae	–	CAB06427	[25]
HCT2	malic acid *O*-hydroxycinnamoyl transferase	*Trifolium pratense*	Viridiplantae	–	ACI16631	[26]
HHT1	omega-hydroxypalmitic acid *O*-feruloyl transferase	*Arabidopsis thaliana*	Viridiplantae	AT5G41040	AED94628	[27]
ligL	β-aryl ether Cα-dehydrogenase	*Sphingobium* sp. SYK-6	Bacteria	SLG_33660	BAK68041	[28]
OsHCT4	glycerol *O*-hydroxycinnamoyltransferase	*Oryza sativa*	Viridiplantae	OSNPB_060185500	BAS96510	[29]
padC	phenolic acid decarboxylase	*Bacillus subtilis* 168	Bacteria	BSU34400	CAB15445	[30]
pcaHG	protocatechuic acid 3,4-dioxygenase	*Stenotrophomonas maltophilia* KB2	Bacteria	–	AFH89645 (*pcaH*)<br>AFH89644 (*pcaG*)	[31]
pcaH2G2	protocatechuic acid 3,4-dioxygenase	*Hydrogenophaga intermedia*	Bacteria	BN948_05072<br>BN948_05073	AAK84297 (*pcaH2*)<br>AAK84298 (*pcaG2*)	[32]
pdc	*p*-coumaric acid decarboxylase	*Lactobacillus plantarum*	Bacteria	LPU63827	AAC45282	[33]
PFOMT	phenylpropanoid/flavonoid *O*-methyltransferase	*Mesembryanthemum crystallinum*	Viridiplantae	–	AAN61072	[34]
PKS1	*p*-coumaroyl-diketide-CoA synthase	*Wachendorfia thyrsiflora*	Viridiplantae	–	AAW50921	[35]
RAS	rosmarinic acid synthase	*Melissa officinalis*	Viridiplantae	–	CBW35684	[36]
rpaI	acyl-homoserine lactone synthases	*Rhodopseudomonas palustris* CGA009	Bacteria	RPA0320	CAE25764	[37]
SDT	spermidine disinapoyl acyltransferase	*Arabidopsis thaliana*	Viridiplantae	AT2G23510	AEC07460	[38]
slr0095	caffeoyl-CoA *O*-methyltransferase	*Synechocystis* sp. PCC 6803	Bacteria	SYNGTS_2605	BAK51353	[39]
SNG1	sinapoylglucose:malic acid *O*-sinapoyltransferase	*Arabidopsis thaliana*	Viridiplantae	AT2G22990	AEC07390	[40]
SPC4	cationic peroxidase	*Sorghum bicolor*	Viridiplantae	LOC8074114	EES02106	[41]
STS2	resveratrol synthase	*Vitis vinifera*	Viridiplantae	VIT_00010554001	ABC84860	[42]
THT10	tyramine hydroxycinnamoyltransferase	*Nicotiana tabacum*	Viridiplantae	LOC107822189	CAB55502	[43]
tyrB	tyrosine aminotransferase	*Escherichia coli* K-12	Bacteria	b4054	NP_418478	[44]
UGT72E1	coniferaldehyde UDP-glucosyl transferase	*Arabidopsis thaliana col*	Viridiplantae	AT3G50740	AEE78703	[45]
UGT84A2	sinapic acid-1-glucosyltransferase	*Arabidopsis thaliana*	Viridiplantae	AT3G21560	AEE76523	[46]
VpVAN	vanillin synthase	*Vanilla planifolia*	Viridiplantae	–	AKG47593	[47]
ydiB	quinic acid/shikimic acid 5-dehydrogenase	*Escherichia coli* K-12	Bacteria	b1692	NP_416207	[48]

Table S2. Summary of enzyme properties for the conversion of metabolites in the shikimate, general phenylpropanoid, flavonoid, or monolignol biosynthetic pathways.

Reaction scheme, coenzyme requirement, enzyme activity (kinetic parameters, specific activity, or substrate range), and reference(s) are shown.

Gene	*F5H*
Enzyme	ferulic acid 5-hydroxylase
Reaction	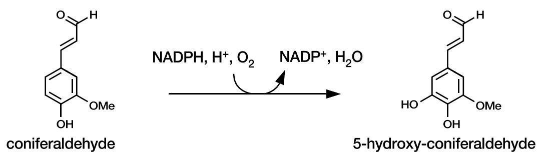
Coenzyme	NADPH
Kinetics<br>V_{max} and K_m	coniferaldehyde, 5 pkat·mg^{-1} and 1 (μM)<br>coniferyl alcohol, 6 pkat·mg^{-1} and 3 (μM)<br>ferulic acid, 4 pkat·mg^{-1} and 1,000 (μM)
Reference	[1]

Gene	*3AT2*
Enzyme	anthocyanin acyltransferase
Reaction	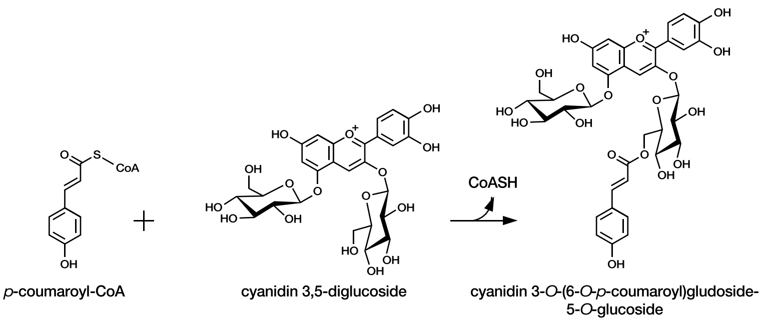
Coenzyme	unnecessary
Kinetics<br>k_{cat} and K_m	*p*-coumaroyl-CoA, 6.9 sec^{-1} and 6.6 μM<br>feruloyl-CoA, 7.7 sec^{-1} and 9.4 μM<br>caffeoyl-CoA, 8.5 sec^{-1} and 4.9 μM<br>sinapoyl-CoA, not determined
Reference	[2]

Gene	*ACT*
Enzyme	agmatine coumaroyltransferase
Reaction	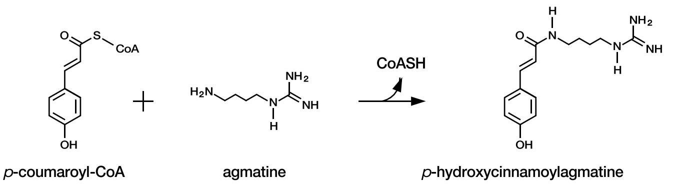
Coenzyme	unnecessary
Specific activity and K_m	*p*-coumaroyl-CoA, 9.9 kat·M^{-1}·g^{-1} and 1.0 μM<br>feruloyl-CoA, 1.4 kat·M^{-1}·g^{-1} and 6.1 μM<br>caffeoyl-CoA, 5.9 kat·M^{-1}·g^{-1} and 5.8 μM
Reference	[3]

Gene	*AT208*
Enzyme	anthocyanidin 3-*O*-glucoside 6'-*O*-acyltransferase
Reaction	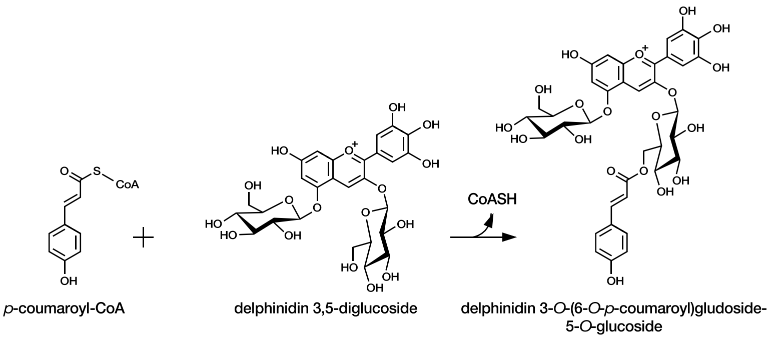
Coenzyme	unnecessary
Kinetics<br>V_{max} and K_{m}	*p*-coumaroyl-CoA, 138 nkat·mg^{-1} and 24 μM<br>caffeoyl-CoA, 168 nkat·mg^{-1} and 45 μM
Reference	[49]

Gene	*Atu1417*
Enzyme	enoyl-CoA hydratase
Reaction	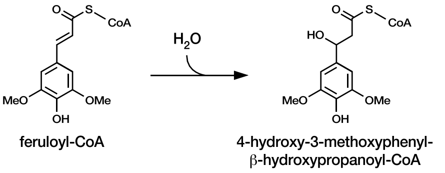
Coenzyme	unnecessary
Substrate range	feruloyl-CoA, *p*-coumaroyl-CoA, caffeoyl-CoA
Reference	[5]

Gene	*Balat_0669*
Enzyme	chlorogenic acid esterase
Reaction	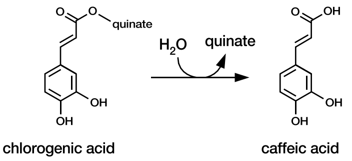
Coenzyme	unnecessary
Specific activity	chlorogenic acid, 2.5 μmol·min^{-1}·mg^{-1}
Reference	[6]

Gene	*BrRCD*
Enzyme	caffeic acid 4,5-dioxygenase
Reaction	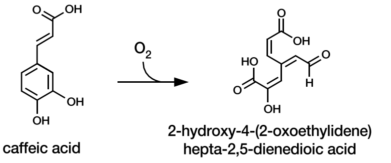
Coenzyme	unnecessary
Substrate range	caffeic acid, homoprotocatechuic acid, protocatechuic acid, catechol
Reference	[7]

Gene	*C2H*
Enzyme	*p*-coumaroyl 2'-hydroxylase
Reaction	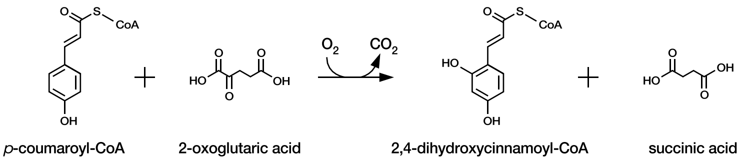
Coenzyme	ascorbate
Kinetics<br>k_{cat} and K_m	*p*-coumaroyl-CoA, 0.64 sec^{-1} and 15.25 μM<br>feruloyl-CoA, 0.55 sec^{-1} and 14.06 μM
Reference	[8]

Gene	*CA9OMT*
Enzyme	coniferyl alcohol 9-*O*-methyltransferase
Reaction	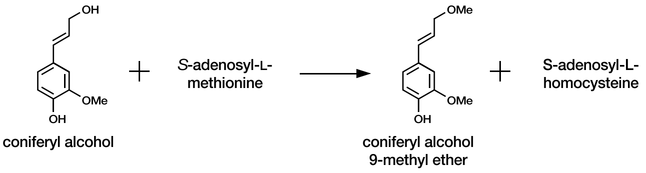
Coenzyme	unnecessary
Kinetics<br>k_{cat} and K_m	coniferyl alcohol, 0.029 sec^{-1} and 7.23 μM
Reference	[9]

Gene	*CADH*
Enzyme	coniferyl alcohol dehydrogenase
Reaction	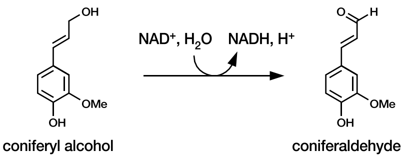
Coenzyme	NAD^+
Kinetics<br>k_{cat} and K_m	coniferyl alcohol, 69.7 sec^{-1} and 130 μM<br>coniferyl alcohol, 116 sec^{-1} and 1700 μM
Reference	[10, 50]

Gene	*CALipB*
Enzyme	lipase B
Reaction	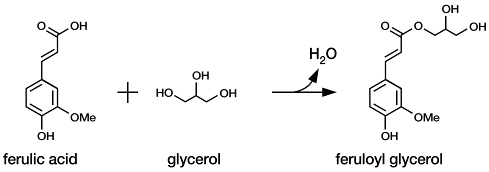
Coenzyme	unnecessary
Substrate range	ferulic acid, *p*-coumaric acid, caffeic acid
Reference	[51, 52]

Gene	*calA*
Enzyme	coniferyl alcohol dehydrogenase
Reaction	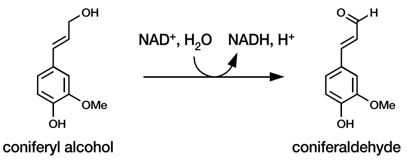
Coenzyme	NAD^+
Substrate	Only reported for coniferyl alcohol
Reference	[12]

Gene	*calB*
Enzyme	coniferyl aldehyde dehydrogenase
Reaction	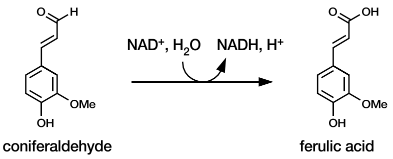
Coenzyme	NAD^+
Specific activity and K_m	coniferaldehyde, 36.5 μmol·min^{-1}·g^{-1} and 7–12 μM<br>sinapaldehyde, 28.1 μmol·min^{-1}·g^{-1}
Reference	[13]

Gene	*CASHT*
Enzyme	hydroxycinnamoyl-CoA: serotonin *N*-(hydroxycinnamoyl)transferase
Reaction	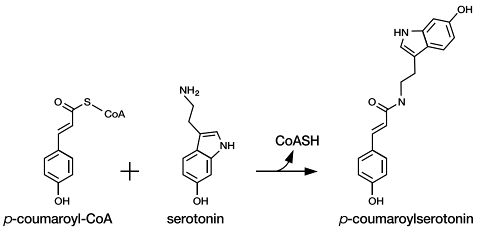
Coenzyme	unnecessary
Kinetics<br>V_{max} and K_m	*p*-coumaroyl-CoA, 35 nkat·mg^{-1} and 23.2 μM<br>feruloyl-CoA, 7 nkat·mg^{-1} and 3.5 μM)<br>caffeoyl-CoA, 31 nkat·mg^{-1} and 12.7 μM
Reference	[14, 53]

Gene	*CCMT1*
Enzyme	cinnamic acid/*p*-coumaric acid carboxyl methyltransferase
Reaction	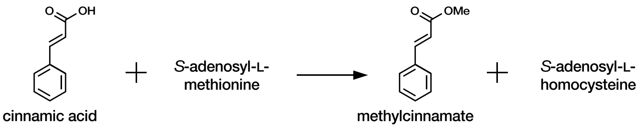
Coenzyme	unnecessary
Kinetics<br>k_{cat} and K_m	cinnamic acid, 0.188 sec^{-1} and 124 μM<br>*p*-coumaric acid, 116 sec^{-1} and 70.4 μM
Reference	[15]

Gene	*CGT*
Enzyme	chlorogenic acid: glucaric acid caffeoyltransferase
Reaction	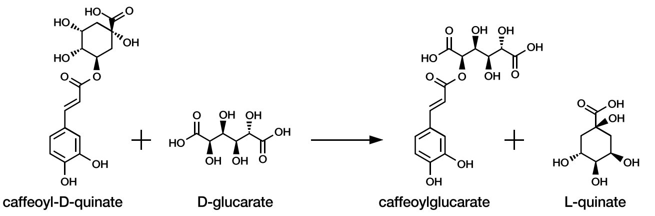
Coenzyme	unnecessary
Kinetics<br>V_{max} and K_{m}	D-glucaric acid, 624 nkat·mg^{-1} and 400 μM<br>D-galactaric acid, 310 nkat·mg^{-1} and 1,700 μM
Reference	[16, 54]

Gene	*CHS5*
Enzyme	chalcone synthase
Reaction	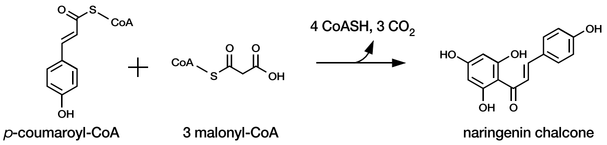
Coenzyme	unnecessary
Substrate range	*p*-coumaroyl-CoA, caffeoyl-CoA, feruloyl-CoA
Reference	[17, 55]

Gene	*couA*
Enzyme	hydroxycinnamoyl-CoA hydratase-lyase
Reaction	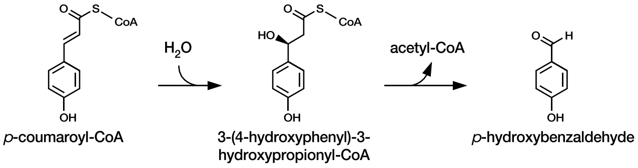
Coenzyme	unnecessary
Substrate range	*p*-coumaroyl-CoA, caffeoyl-CoA, feruloyl-CoA
Reference	[18]

Gene	*CURS3*
Enzyme	curcumin synthase
Reaction	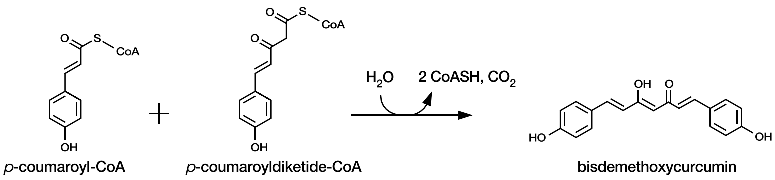
Coenzyme	unnecessary
Kinetics<br>k_{cat} and K_{m}	*p*-coumaroyl-CoA, 0.006 sec^{-1} and 3.4 μM<br>(*p*-coumaroyl-CoA + cinnamoyldiketide-*N*-acetylcysteamine)<br>feruloyl-CoA, 0.003 sec^{-1} and 2.2 μM<br>(feruloyl-CoA + cinnamoyldiketide-*N*-acetylcysteamine)
Reference	[19]

Gene	*CYP84A4*
Enzyme	*p*-coumaraldehyde 3-hydroxylase
Reaction	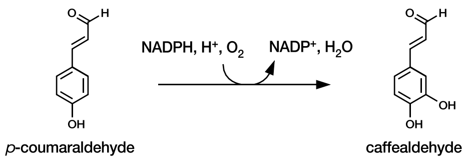
Coenzyme	NADPH
Kinetics<br>V_{max} and K_m	*p*-coumaraldehyde, 0.69 pkat·mg^{-1} and 41 μM
Reference	[20]

Gene	*CYP98A22*
Enzyme	*p*-coumaroyl 2'-hydroxylase
Reaction	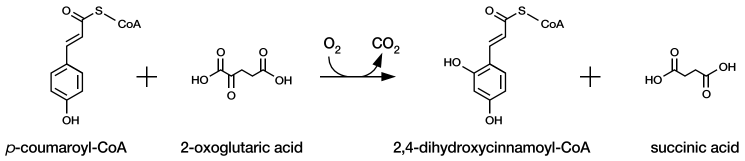
Coenzyme	ascorbate
Kinetics<br>k_{cat} and K_m	*p*-coumaroyl-CoA, 0.71 sec^{-1} and 50 μM<br>feruloyl-CoA, 0.46 sec^{-1} and 37 μM
Reference	[21]

Gene	*F6H1*
Enzyme	feruloyl-CoA 6′-hydroxylase
Reaction	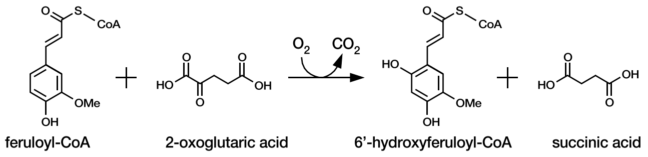
Cofactor	ascorbate
Kinetics<br>k_{cat} and K_m	feruloyl-CoA, 11.0 sec^{-1} and 36 μM<br>*p*-coumaroyl-CoA, trace reaction
Reference	[22]

Gene	*FDC1*
Enzyme	phenacrylic acid decarboxylase
Reaction	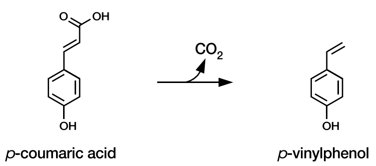
Cofactor	prenylated $FMNH_2$
Kinetics<br>k_{cat} and K_m	*p*-coumaric acid, 1.5 sec^{-1} and 110 μM<br>cinnamic acid, 4.6 sec^{-1} and 180 μM<br>ferulic acid, 3.8 sec^{-1} and 180 μM
Reference	[56]

Gene	*HCALDH*
Enzyme	hydroxycinnamaldehyde dehydrogenase
Reaction	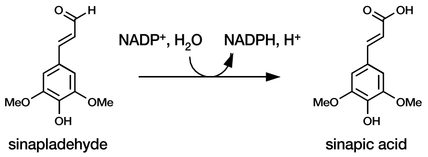
Coenzyme	$NADP^+$
Kinetics<br>V_{max} and K_m	coniferaldehyde, 560 pkat·mg^{-1} and 43 μM<br>sinapaldehyde, 1700 pkat·mg^{-1} and 150 μM<br>*p*-coumarladehyde, 310 pkat·mg^{-1} and 19 μM
Reference	[57]

Gene	*HCBT*
Enzyme	anthranilic acid *N*-hydroxycinnamoyl/benzoyltransferase
Reaction	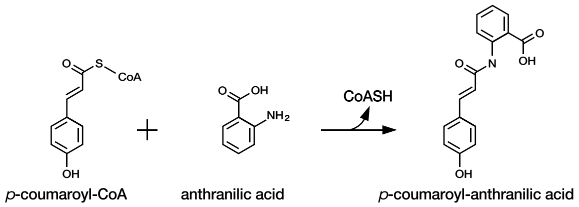
Coenzyme	unnecessary
Substrate range	*p*-coumaroyl-CoA, caffeoy-CoA, feruloyl-CoA, sinapoyl-CoA
Reference	[25, 58]

Gene	*HCT2*
Enzyme	malic aicd *O*-hydroxycinnamoyl transferase
Reaction	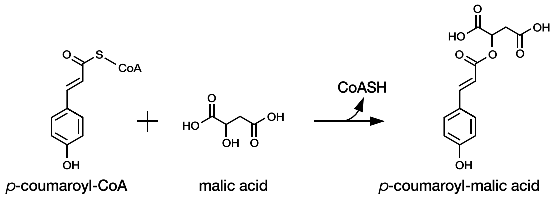
Coenzyme	unnecessary
Specific activity	*p*-coumaroyl-CoA, 34 nkat·mg^{-1}<br>caffeoyl-CoA, 5.1 nkat·mg^{-1}
Reference	[26]

Gene	*HHT1*
Enzyme	omega-hydroxypalmitic acid *O*-feruloyl transferase
Reaction	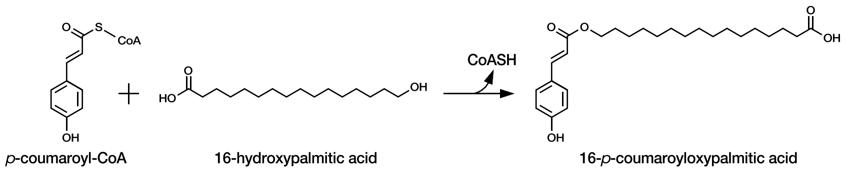
Coenzyme	unnecessary
Kinetics<br>k_{cat} and K_m	*p*-coumaroyl-CoA, 0.0136 sec^{-1} and 36.7 μM<br>feruloyl-CoA, 0.0173 sec^{-1} and 9.7 μM
Reference	[27]

Gene	*ligL*
Enzyme	β-aryl ether Cα-dehydrogenase
Reaction	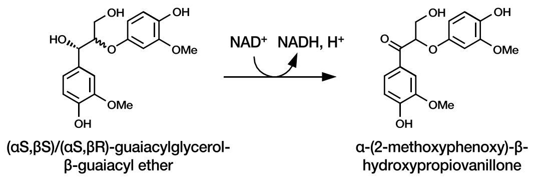
Coenzyme	NAD^+
Substrate range	β-O-4 dimers and oligomers with guaiacyl and syringyl structure
Reference	[28, 59]

Gene	*OsHCT4*
Enzyme	glycerol *O*-hydroxycinnamoyltransferase
Reaction	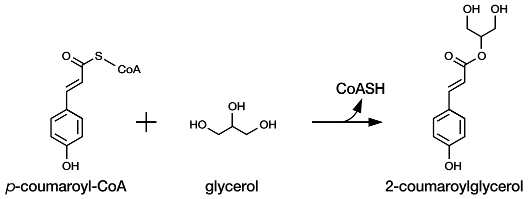
Coenzyme	unnecessary
Kinetics<br>k_{cat} and K_m	*p*-coumaroyl-CoA, 0.037 sec^{-1} and 60 μM<br>feruloyl-CoA, 0.071 sec^{-1} and 72 μM<br>caffeoyl-CoA, 0.082 sec^{-1} and 102 μM
Reference	[29]

Gene	*padC*
Enzyme	phenolic acid decarboxylase
Reaction	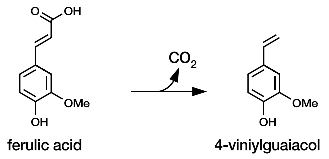
Coenzyme	prenylated $FMNH_2$?
Kinetics<br>V_{max} and K_m	ferulic acid, 280 $\mu mol \cdot min^{-1} \cdot mg^{-1}$ and 1100 μM<br>*p*-coumaric acid, 265 $\mu mol \cdot min^{-1} \cdot mg^{-1}$ and 1300 μM<br>caffeic acid, 180 $\mu mol \cdot min^{-1} \cdot mg^{-1}$ and 2600 μM
Reference	[30]

Gene	*pcaG* + *pcaH*
Enzyme	protocatechuic acid 3,4-dioxygenase
Reaction	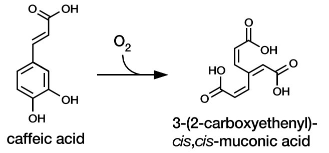
Coenzyme	unnecessary
Substrate range	caffeic acid and dihydrocaffeic acid
Reference	[31]

Gene	*pcaG2 + pcaH2*
Enzyme	protocatechuic acid 3,4-dioxygenase
Reaction	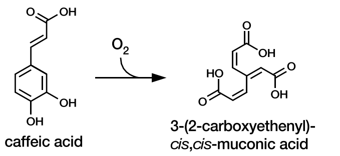
Coenzyme	unnecessary
Substrate range	caffeic acid and 3,4-dihydroxyphenylacetic acid
Reference	[32]

Gene	*pdc*
Enzyme	*p*-coumaric acid decarboxylase
Reaction	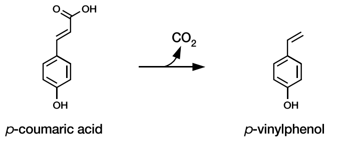
Coenzyme	prenylated $FMNH_2$
Kinetics V_{max} and K_m	*p*-coumaric acid, 766 μmol·min^{-1}·mg^{-1} and 1400 μM
Reference	[33]

Gene	*PFOMT*
Enzyme	phenylpropanoid/flavonoid *O*-methyltransferase
Reaction	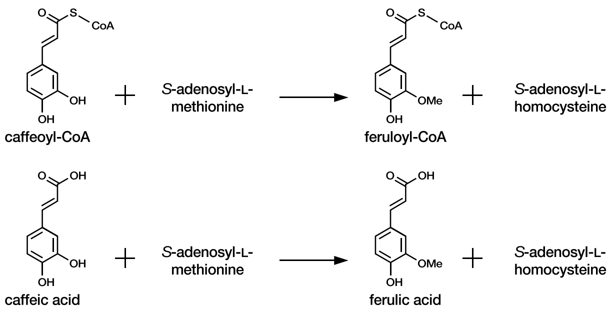
Coenzyme	unnecessary
Kinetics V_{max} and K_m	caffeoyl-CoA, 0.025 μmol·min^{-1}·mg^{-1} and 25 μM<br>caffeic acid, 0.005 μmol·min^{-1}·mg^{-1} and 44 μM<br>quercetin, 0.034 μmol·min^{-1}·mg^{-1} and 6.4 μM
Reference	[34]

Gene	*PKS1*
Enzyme	*p*-coumaroyl-diketide-CoA synthase
Reaction	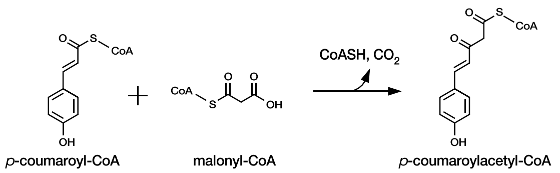
Coenzyme	unnecessary
Kinetics k_{cat} and K_m	*p*-coumaroyl-CoA, 0.00048sec^{-1} and 85 μM<br>phenylpropionyl-CoA, 0.0025 sec^{-1} and 40 μM<br>cinnamoyl-CoA, 0.00053 sec^{-1} and 28 μM
Reference	[35]

Gene	*RAS*
Enzyme	rosmarinic acid synthase
Reaction	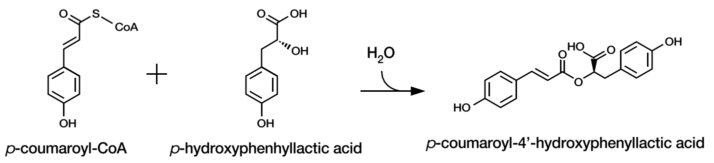
Coenzyme	unnecessary
Kinetics V_{max} and K_m	*p*-coumaroyl-CoA (+*p*-hydroxyphenhyllactic acid), 1076 pkat·mg^{-1} and 9.4 μM<br>*p*-coumaroyl-CoA (+3,4-dihydroxyphenhyllactic acid), 294 pkat·mg^{-1} and 3.2 μM<br>caffeoyl-CoA (+*p*-hydroxyphenhyllactic acid), 283 pkat·mg^{-1} and 2.3 μM<br>caffeoyl-CoA (+3,4-dihydroxyphenhyllactic acid), 103 pkat·mg^{-1} and 3.3 μM
Reference	[37]

Gene	*rpaI*
Enzyme	acyl-homoserine lactone synthases
Reaction	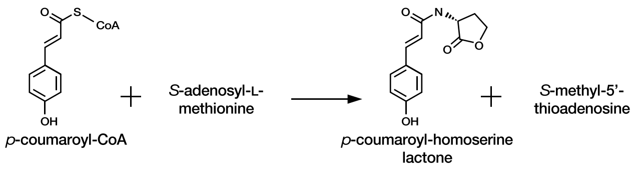
Coenzyme	unnecessary
Reference	[36]

Gene	*SDT*
Enzyme	spermidine disinapoyl acyltransferase
Reaction	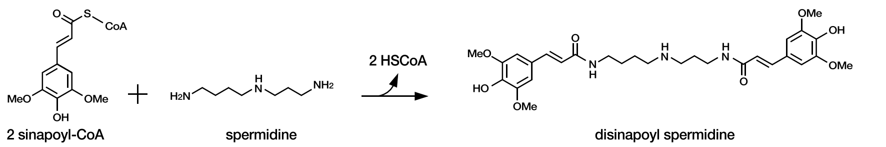
Coenzyme	unnecessary
Kinetics k_{cat} and K_m	sinapoyl-CoA, 5.1 sec^{-1} and 8.3 μM
Reference	[38]

Gene	*slr0095*
Enzyme	caffeoyl-CoA *O*-methyltransferase
Reaction	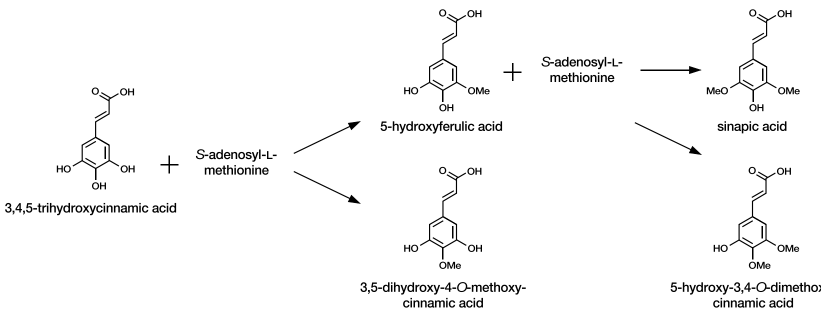
Coenzyme	unnecessary
Kinetics<br>k_{cat} and K_m	3,4,5-tryhydroxycinnamic acid, 0.0104 liter·sec^{-1} and 20.7 μM<br>caffeoyl-CoA, 0.0245 liter·sec^{-1} and 32.9 μM<br>caffeic acid, 0.0055 liter·sec^{-1} and 69.3 μM<br>5-hydroxyferulic acid, 0.0250 liter·sec^{-1} and 74.0 μM
Reference	[39]

Gene	*SNG1*
Enzyme	sinapoylglucose:malic acid *O*-sinapoyltransferase
Reaction	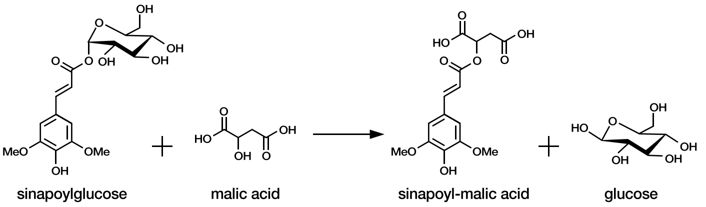
Coenzyme	unnecessary
Kinetics<br>V_{max} and K_m	sinapoylglucose, 0.528 μmol·min^{-1}·mg^{-1} and 460 μM
Reference	[40]

Gene	*SPC4*
Enzyme	cationic peroxidase
Reaction	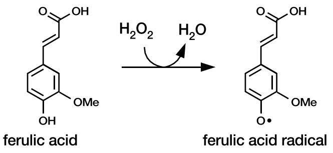
Coenzyme	unnecessary
Kinetics<br>V_{max} /K_m	ferulic acid, 0.92 M^{-1}·s^{-1}<br>*p*-coumaric acid, 0.23 M^{-1}·s^{-1}
Reference	[41]

Gene	*STS2*
Enzyme	resveratrol synthase
Reaction	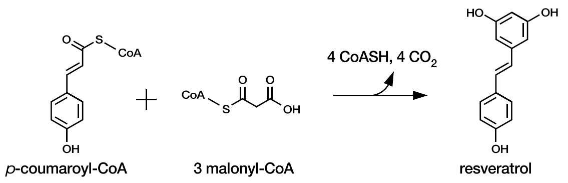
Coenzyme	unnecessary
Reference	[42]

Gene	*THT10*
Enzyme	tyramine hydroxycinnamoyltransferase
Reaction	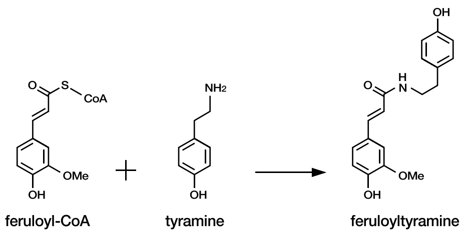
Coenzyme	unnecessary
Kinetics<br>V_{max} and K_m	feruloyl-CoA, 69960 pkat·mg^{-1} and 0.6 μM<br>sinapoyl-CoA, 76000 pkat·mg^{-1} and 1 μM<br>*p*-coumaroyl-CoA, 19200 pkat·mg^{-1} and 2 μM<br>cinnamoyl-CoA, 120320 pkat·mg^{-1} and 4.7 μM
Reference	[43]

Gene	*tyrB*
Enzyme	tyrosine aminotransferase
Reaction	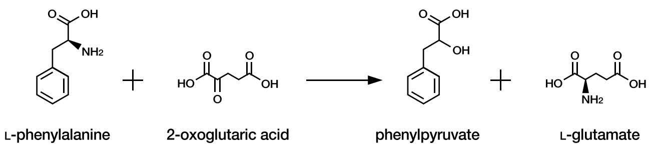
Coenzyme	unnecessary
Kinetics<br>V_{max} and K_m	L-phenylalanine, 9.1 μmol·min^{-1}·nmol protein^{-1} and 333 μM<br>L-tyrosine, 20.0 μmol·min^{-1}·nmol protein^{-1} and 625 μM
Reference	[44]

Gene	*UGT72E1*
Enzyme	coniferaldehyde UDP-glucosyl transferase
Reaction	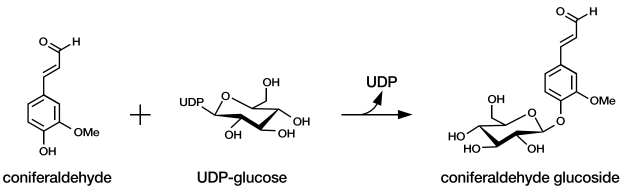
Coenzyme	unnecessary
Kinetics<br>k_{cat} and K_m	coniferaldehyde, 1.22 sec^{-1} and 270 μM<br>sinapaldehyde, 1.43 sec^{-1} and 460 μM
Reference	[45]

Gene	*UGT84A2*
Enzyme	sinapic acid-1-glucosyltransferase
Reaction	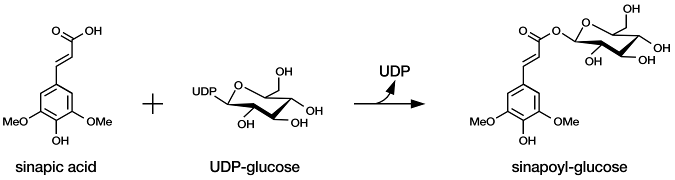
Coenzyme	unnecessary
Kinetics<br>V_{max} and K_m	sinapic acid, 13.44 nkat·mg^{-1} and 250 μM
Reference	[46]

Gene	*VpVAN*
Enzyme	vanillin synthase
Reaction	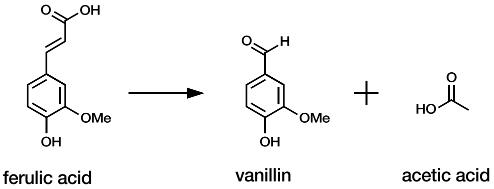
Coenzyme	unnecessary
Substrate range	ferulic acid and ferulic acid glucoside
Reference	[47]

Gene	*ydiB*
Enzyme	quinic acid/shikimic acid 5-dehydrogenase
Reaction	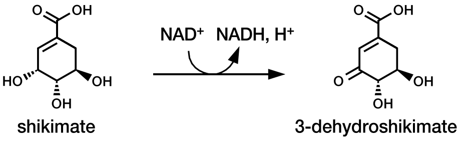
Coenzyme	NAD^+
Kinetics<br>k_{cat} and K_m	shikimic acid, 91000 sec^{-1} and 2.9 μM<br>quinic acid, 113000 sec^{-1} and 9.1 μM
Reference	[48]

Table S3. List of plants generated and mixed Agrobacterium strains harboring different gene constructs used for seven independent inoculation procedures.

Gene set		Gene	Number of plants	Efficiency (%)
1	Single-gene introduction	*CASHT*	9	18.4
		pdc	7	14.3
		CCMT1	7	14.3
		tyrB	6	12.2
		THT10	5	10.2
		FDC1	4	8.2
		couA	2	4.1
		Atu1417	0	0
	Multiple-genes introduction		9	-
	Subtotal		49	-
2	Single-gene introduction	*rpaI*	11	26.2
		STS2	7	16.7
		C2H	6	14.3
		CURS3	2	4.8
		PKS1	2	4.8
		AT208	2	4.8
		CHS5	1	2.4
	Multiple-genes introduction		11	-
	Subtotal		42	-
3	Single-gene introduction	*3AT2*	11	22.4
		HCBT	8	16.3
		ACT	6	12.2
		HCT2	5	10.2
		OsHCT4	5	10.2
		RAS	2	4.1
		SDT	2	4.1
		CYP84A4	2	4.1
	Multiple-genes introduction		8	-
	Subtotal		49	-
4	Single-gene introduction	*CALipB*	8	16.3
		SPC4	8	16.3
		UGT84A2	6	12.2
		BrRCD	4	8.2
		F5H	4	8.2
		PFOMT	3	6.1
		slr0095	2	4.1
		VpVAN	2	4.1
	Multiple-genes introduction		12	-
	Subtotal		49	-

Table S3. ***continued***

Gene set	Gene		Number of Plants	Efficiency (%)
5	Single-gene introduction	*Balat_0669*	8	16.3
		calB	8	16.3
		HHT1	5	10.2
		HCALDH	5	10.2
		CGT	4	8.2
		F6H1	4	8.2
		UGT72E1	4	8.2
		padC	0	0
	Multiple-genes introduction		11	-
	Subtotal		49	-
6	Single-gene introduction	*CYP98A22*	7	16.3
		CA9OMT	6	14
		CADH	3	9.3
		calA	4	9.3
		ligL	2	4.7
		ydiB	2	4.7
		SNG1	2	4.7
	Multiple-genes introduction		16	-
	Subtotal		43	-
7	Single-gene introduction	*pcaG2 + pcaH2*	8	53.3
		pcaG + pcaH	7	46.7
	Multiple-genes introduction		0	-
	Subtotal		15	-

The number of transgenic plants generated with a single gene and with multiple transgenes are indicated in the third column. Efficiency refers to the relative proportions of plants harboring a particular gene among all of the plants generated with the same mixture of Agrobacterium strains.

Table S4. Relative abundance of pyrolysis products within AIR-sample from wild-type and transgenic inflorescence stem

No.	Products	Stractural feature	Wild type	*PkC4Hpro:: F5H*	*PkC4Hpro:: calB*	*PkC4Hpro:: couA*	*PkC4Hpro:: F6H1*
1	Phenol	H, unsub	0.8 (0.36)	0.49 (0.26)	0.65 (0.21)	*1.36 (0.24)**	0.81 (0.3)
2	2-methylphenol	H, unsub	0.19 (0.06)	*0.08 (0.05)**	0.16 (0.05)	0.23 (0.04)	0.15 (0.07)
3	4-Methylphenol	H, unsub	0.41 (0.14)	*0.21 (0.12)**	0.36 (0.07)	*0.65 (0.1)**	0.42 (0.15)
4	Guaiacol	G, unsub	2.87 (0.56)	*0.56 (0.49)****	2.73 (0.2)	2.79 (0.31)	2.56 (0.38)
5	hydroquinone	H, misc	1.4 (0.44)	0.85 (0.52)	1.4 (0.36)	1.98 (0.48)	1.55 (0.5)
6	4-ethylphenol	H, ethyl	ND	ND	ND	0.01 (0.01)	0.01 (0.02)
7	2,4-dimethylphenol	H, methyl	1.12 (0.14)	*0.22 (0.25)****	1.13 (0.15)	1.1 (0.17)	1.26 (0.22)
8	4-methylguaiacol	G, methyl	1.36 (0.14)	*0.25 (0.3)****	1.35 (0.16)	1.29 (0.18)	1.52 (0.27)
9	4-Ethylguaiacol	G, ethyl	0.15 (0.08)	ND	0.15 (0.09)	*0.31 (0.05)***	0.15 (0.09)
10	4-Vinylguaiacol	G, vinyl	12.13 (2.15)	*2.34 (2.26)****	11.79 (0.74)	*15.45 (1.71)**	11.43 (1.74)
11	4-vinylphenol	H, vinyl	8.09 (1.43)	*1.56 (1.51)****	7.86 (0.49)	*10.3 (1.14)**	7.62 (1.16)
12	3-methoxy-5-methylphenol	G, methyl	0.02 (0.02)	0.03 (0.02)	0.04 (0.02)	*0.08 (0.05)**	0.03 (0.03)
13	4-propenylphenol	H, methyl	0.02 (0.01)	0.02 (0.01)	0.01 (0.01)	0.03 (0.01)	0.02 (0.02)
14	syringol	S, unsub	1.38 (0.26)	*3.51 (0.7)****	*1.96 (0.32)***	*1.99 (0.39)***	*1.79 (0.35)**
15	Eugenol	G, misc	0.61 (0.08)	*0.13 (0.14)****	0.59 (0.07)	*0.41 (0.07)***	0.64 (0.12)
16	4-hydroxybenzaldehyde	H, Cα-O	0.03 (0.01)	0.02 (0.01)	0.02 (0.02)	0.06 (0.04)	0.03 (0.03)
17	4-Propylguaiacol	G, misc	0.27 (0.05)	*0.05 (0.07)****	0.27 (0.05)	*0.33 (0.04)**	0.28 (0.05)
18	Vanillin	G, Cα-O	1.01 (0.11)	*0.21 (0.17)****	0.93 (0.12)	0.88 (0.07)	0.98 (0.13)
19	cis-isoeugenol	G, misc	0.49 (0.1)	*0.11 (0.11)****	0.47 (0.07)	0.38 (0.05)	0.5 (0.1)
20	4-methylsyringol	S, methyl	0.71 (0.13)	*1.46 (0.29)****	*1.01 (0.15)***	*1.08 (0.12)****	*1.11 (0.14)****
21	trans-Isoeugenol	G, misc	3.4 (0.64)	*0.76 (0.76)****	3.32 (0.37)	*2.6 (0.31)**	3.5 (0.67)
22	4-propyneguaiacol	G, misc	0.35 (0.06)	*0.16 (0.05)****	0.31 (0.06)	0.29 (0.06)	0.32 (0.08)
23	4-alleneguaiacol	G, misc	0.31 (0.06)	*0.14 (0.04)***	0.27 (0.06)	0.25 (0.05)	0.28 (0.06)
24	acetovanilone	G, Cα-O	1.08 (0.03)	*0.25 (0.2)****	*0.99 (0.05)***	*1.34 (0.15)***	1.06 (0.13)
25	4-ethylsyringol	S, ethyl	0.05 (0.01)	*0.12 (0.04)***	*0.08 (0.02)**	*0.11 (0.02)****	*0.07 (0.02)**
26	Guaiacylaceton	G, Cβ-O	1.21 (0.11)	*0.25 (0.27)****	1.19 (0.04)	1.1 (0.06)	1.34 (0.21)
27	4-vinlysyringol	S, vinyl	5.29 (0.73)	*13.16 (2.94)****	*7.22 (1.28)***	*9.26 (0.82)****	*6.87 (1.05)***
28	4-allylsyringol	S, misc	0.42 (0.06)	*1.2 (0.35)****	*0.6 (0.1)***	*0.65 (0.09)****	*0.62 (0.08)****
29	homosyringaldehyde	S, Cβ-O	0.08 (0.01)	*0.18 (0.05)****	*0.11 (0.02)**	*0.13 (0.02)****	*0.11 (0.02)***
30	cis-4-propenylsyringol	S, misc	0.29 (0.05)	*0.8 (0.25)****	*0.41 (0.08)***	*0.49 (0.07)****	*0.42 (0.07)***
31	Syringaldehyde	S, Cα-O	0.9 (0.13)	*2.8 (0.34)****	*1.29 (0.15)****	1.05 (0.22)	*1.29 (0.18)****
32	cis-Coniferyl-alcohol	G, Cc-O	3.45 (0.18)	*0.69 (0.8)****	*3.14 (0.27)**	*2.45 (0.24)****	*3.15 (0.27)**
33	4-propynesyringol	S, misc	0.22 (0.03)	*0.76 (0.27)****	*0.31 (0.05)****	*0.34 (0.05)****	*0.29 (0.05)***
34	4-allenesyringol	S, misc	0.16 (0.02)	*0.52 (0.18)****	*0.22 (0.04)***	*0.24 (0.04)****	*0.2 (0.03)**
35	trans-4-propenylsyringol	S, misc	2 (0.35)	*5.55 (1.71)****	*2.85 (0.53)***	*3.36 (0.47)****	*2.89 (0.44)***
36	acetosyringone	S, Cα-O	0.11 (0.15)	*1.06 (0.32)****	0.25 (0.19)	*0.36 (0.23)**	*0.35 (0.15)**
37	Coniferylaldehyde	G, Cγ-O	1.12 (0.88)	ND	0.54 (0.78)	0.16 (0.3)	1.27 (0.74)
38	trans-coniferyl-alcohol	G, Cγ-O	30.77 (2.46)	*7.07 (5.66)****	*26.41 (3.03)**	*19.46 (1.83)****	*24.87 (3.24)***
39	Syringylacetone	S, Cβ-O	0.17 (0.02)	*0.54 (0.12)****	*0.24 (0.04)****	*0.32 (0.04)****	*0.25 (0.05)****
41	Coniferylacetate	S, Cγ-O	0.01 (0)	ND	0.01 (0)	*0.01 (0.01)**	*0.02 (0)**
42	cis-Sinapyl alcohol	S, Cγ-O	1.22 (0.19)	*3.72 (0.49)****	*1.54 (0.11)**	1.45 (0.25)	*1.6 (0.25)***
43	trans-sinapalaldehyde	S, Cγ-O	0.08 (0.09)	*0.65 (0.49)**	0.08 (0.12)	0.02 (0.03)	0.13 (0.09)
44	trans-sinapyl-alcohol	S, Cγ-O	14.26 (3.74)	*47.49 (11.44)****	15.73 (1.78)	13.81 (2.95)	15.61 (4.53)

Peak molar areas were calculated for the lignin degradation products with relative responce factor reporte by van Erven (2017).

The summed areas were normalized, and the amount of each compound is expressed as percentages.

Numbers in parentheses are 95% confidential intervals.

Table S5. carbohydrates composition in transgenic lines supporting Fig.6

Lines	Carbohydrates (µg/mgAIR)										Total carbohydrates (µg/mgAIR)
	Glc	Xyl	GalA	Rha	Man	Gal	Ara	GlcA	mGlcA	Fuc	
Wild-type	377 (16)	145 (7)	119 (9)	44.9 (2.9)	30.5 (1.1)	22.1 (2)	12.7 (2.2)	5.92 (0.43)	2.03 (0.27)	1.07 (0.11)	685 (26)
PkC4Hpro::F5H	381 (9)	*154 (6)**	120 (11)	46 (3.7)	31.8 (1.4)	23.4 (1.9)	14.5 (1.7)	6.48 (0.78)	2.09 (0.86)	1.08 (0.21)	702 (41)
PkC4Hpro::calB	377 (10)	151 (5)	130 (9)	48 (2.9)	31 (1.4)	*24.2 (1.3)**	15.1 (1.6)	5.91 (0.52)	*5.92 (2.28)****	1.11 (0.19)	710 (34)
PkC4Hpro::couA	*326 (10)****	143 (6)	*140 (6)***	*63.9 (5.1)****	31.2 (1.1)	*36 (3.7)****	*31.8 (6.3)****	*6.8 (0.55)***	*7.53 (2.75)****	1.21 (0.09)*	708 (23)
PkC4Hpro::F6H1	*352 (18)**	*133 (8)**	128 (7)	46.5 (2.2)	*28.3 (1.4)**	23.5 (1.2)	14.2 (1.2)	5.71 (0.55)	1.87 (1.15)	1.23 (0.11)**	661 (50)

Numbers in parentheses are 95% confidential intervals.

Table S6. sugar yield by enzymatic saccharification supporting to Fig.7

Line	Glucose yield (μg/mgAIR)		Xylose yield (μg/mgAIR)	
	Without pretreatment	Pretreatment with 100 mM NaOH	Without pretreatment	Pretreatment with 100 mM NaOH
Wild-type	126.2 (16)	136 (11)	3.9 (2)	87 (5)
PkC4Hpro::F5H	126.1 (12)	*178 (32)**	*0.9 (1)**	*127 (16)****
PkC4Hpro::calB	133.5 (17)	164 (25)	4.5 (2)	*108 (12)***
PkC4Hpro::couA	144.7 (13)	*210 (17)****	*17.9 (7)***	*134 (16)****
PkC4Hpro::F6H1	118.8 (7)	140 (13)	*0.8 (1)**	91 (10)

Numbers in parentheses are 95% confidential intervals.

References

1. Humphreys JM, Hemm MR, Chapple C: New routes for lignin biosynthesis defined by biochemical characterization of recombinant ferulate 5-hydroxylase, a multifunctional cytochrome P450-dependent monooxygenase. *Proceedings of the National Academy of Sciences of the United States of America.* 1999;96(18):10045-10050.

2. Luo J, Nishiyama Y, Fuell C, Taguchi G, Elliott K, Hill L, Tanaka Y, Kitayama M, Yamazaki M, Bailey P *et al*: Convergent evolution in the BAHD family of acyl transferases: identification and characterization of anthocyanin acyl transferases from *Arabidopsis thaliana*. *Plant J.* 2007;50(4):678-695.

3. Burhenne K, Kristensen BK, Rasmussen SK: A new class of *N*-hydroxycinnamoyltransferases. Purification, cloning, and expression of a barley agmatine coumaroyltransferase (EC 2.3.1.64). *The Journal of biological chemistry.* 2003;278(16):13919-13927.

4. Fujiwara H, Tanaka Y, Yonekura-Sakakibara K, Fukuchi-Mizutani M, Nakao M, Fukui Y, Yamaguchi M, Ashikari T, Kusumi T: cDNA cloning, gene expression and subcellular localization of anthocyanin 5-aromatic acyltransferase from *Gentiana triflora*. *Plant J.* 1998;16(4):421-431.

5. Campillo T, Renoud S, Kerzaon I, Vial L, Baude J, Gaillard V, Bellvert F, Chamignon C, Comte G, Nesme X *et al*: Analysis of hydroxycinnamic acid degradation in *Agrobacterium fabrum* reveals a coenzyme A-dependent, β-oxidative deacetylation pathway. *Appl Environ Microbiol.* 2014;80(11):3341-3349.

6. Raimondi S, Anighoro A, Quartieri A, Amaretti A, Tomás‐Barberán F, Rastelli G, Rossi M: Role of bifidobacteria in the hydrolysis of chlorogenic acid. *MicrobiologyOpen.* 2015;4(1):41-52.

7. Kobayashi Y: Screening of bacterial aromatic ring-cleavage dioxygenases for lignin biosynthesis intermediates. *Unpublished Mater's Thesis, Nagaoka University of Technology.* 2016:1-48.

8. Matsumoto S, Mizutani M, Sakata K, Shimizu B: Molecular cloning and functional analysis of the *ortho*-hydroxylases of *p*-coumaroyl coenzyme A/feruloyl coenzyme A involved in formation of umbelliferone and scopoletin in sweet potato, *Ipomoea batatas* (L.) Lam. *Phytochemistry.* 2012;74:49-57.

9. Berim A, Schneider B, Petersen M: Methyl allyl ether formation in plants: novel *S*-adenosyl L-methionine:coniferyl alcohol 9-*O*-methyltransferase from suspension cultures of three *Linum* species. *Plant Mol Biol.* 2007;64(3):279-291.

10. Nishimura M: Molecular cloning and expression of the *Streptomyces* coniferyl alcohol dehydrogenase gene in *Escherichia coli*. *Protein Expr Purif.* 2013;89(1):109-115.

11. Uppenberg J, Hansen MT, Patkar S, Jones TA: The sequence, crystal structure determination and refinement of two crystal forms of lipase B from *Candida antarctica*. *Structure.* 1994;2(4):293-308.

12. Overhage J, Steinbüchel A, Priefert H: Biotransformation of eugenol to ferulic acid by a recombinant strain of *Ralstonia eutropha* H16. *Appl Environ Microbiol.* 2002;68(9):4315-4321.

13. Achterholt S, Priefert H, Steinbuchel A: Purification and characterization of the coniferyl aldehyde dehydrogenase from *Pseudomonas* sp. strain HR199 and molecular characterization of the gene. *J Bacteriol.* 1998;180(17):4387-4391.

14. Back K, Jang SM, Lee BC, Schmidt A, Strack D, Kim KM: Cloning and characterization of a hydroxycinnamoyl-CoA:tyramine *N*-(hydroxycinnamoyl)transferase induced in response to UV-C and wounding from *Capsicum annuum*. *Plant Cell Physiol.* 2001;42(5):475-481.

15. Kapteyn J, Qualley AV, Xie Z, Fridman E, Dudareva N, Gang DR: Evolution of cinnamate/*p*-coumarate carboxyl methyltransferases and their role in the biosynthesis of methylcinnamate. *Plant Cell.* 2007;19(10):3212-3229.

16. Teutschbein J, Gross W, Nimtz M, Milkowski C, Hause B, Strack D: Identification and localization of a lipase-like acyltransferase in phenylpropanoid metabolism of tomato (*Solanum lycopersicum*). *The Journal of biological chemistry.* 2010;285(49):38374-38381.

17. Akada S, Dube SK: Organization of soybean chalcone synthase gene clusters and characterization of a new member of the family. *Plant Mol Biol.* 1995;29(2):189-199.

18. Hirakawa H, Schaefer AL, Greenberg EP, Harwood CS: Anaerobic *p*-coumarate degradation by *Rhodopseudomonas palustris* and identification of CouR, a MarR repressor protein that binds *p*-coumaroyl coenzyme A. *J Bacteriol.* 2012;194(8):1960-1967.

19. Katsuyama Y, Kita T, Horinouchi S: Identification and characterization of multiple curcumin synthases from the herb *Curcuma longa*. *FEBS letters.* 2009;583(17):2799-2803.

20. Weng JK, Li Y, Mo H, Chapple C: Assembly of an evolutionarily new pathway for α-pyrone biosynthesis in *Arabidopsis*. *Science.* 2012;337(6097):960-964.

21. Vialart G, Hehn A, Olry A, Ito K, Krieger C, Larbat R, Paris C, Shimizu B, Sugimoto Y, Mizutani M *et al*: A 2-oxoglutarate-dependent dioxygenase from *Ruta graveolens* L. exhibits *p*-coumaroyl CoA 2'-hydroxylase activity (C2'H): a missing step in the synthesis of umbelliferone in plants. *Plant J.* 2012;70(3):460-470.

22. Kai K, Mizutani M, Kawamura N, Yamamoto R, Tamai M, Yamaguchi H, Sakata K, Shimizu B: Scopoletin is biosynthesized via *ortho*-hydroxylation of feruloyl CoA by a 2-oxoglutarate-dependent dioxygenase in *Arabidopsis thaliana*. *Plant J.* 2008;55(6):989-999.

23. Mukai N, Masaki K, Fujii T, Kawamukai M, Iefuji H: PAD1 and FDC1 are essential for the decarboxylation of phenylacrylic acids in *Saccharomyces cerevisiae*. *J Biosci Bioeng.* 2010;109(6):564-569.

24. Nair RB, Bastress KL, Ruegger MO, Denault JW, Chapple C: The *Arabidopsis thaliana REDUCED EPIDERMAL FLUORESCENCE1* gene encodes an aldehyde dehydrogenase involved in ferulic acid and sinapic acid biosynthesis. *Plant Cell.* 2004;16(2):544-554.

25. Yang Q, Reinhard K, Schiltz E, Matern U: Characterization and heterologous expression of hydroxycinnamoyl/benzoyl-CoA:anthranilate *N*-hydroxycinnamoyl/benzoyltransferase from elicited cell cultures of carnation, *Dianthus caryophyllus* L. *Plant Mol Biol.* 1997;35(6):777-789.

26. Sullivan M: A novel red clover hydroxycinnamoyl transferase has enzymatic activities consistent with a role in phaselic acid biosynthesis. *Plant Physiol.* 2009;150(4):1866-1879.

27. Gou JY, Yu XH, Liu CJ: A hydroxycinnamoyltransferase responsible for synthesizing suberin aromatics in *Arabidopsis*. *Proceedings of the National Academy of Sciences of the United States of America.* 2009;106(44):18855-18860.

28. Sato Y, Moriuchi H, Hishiyama S, Otsuka Y, Oshima K, Kasai D, Nakamura M, Ohara S, Katayama Y, Fukuda M *et al*: Identification of three alcohol dehydrogenase genes involved in the stereospecific catabolism of arylglycerol-β-aryl ether by *Sphingobium* sp. strain SYK-6. *Appl Environ Microbiol.* 2009;75(16):5195-5201.

29. Kim IA, Kim BG, Kim M, Ahn JH: Characterization of hydroxycinnamoyltransferase from rice and its application for biological synthesis of hydroxycinnamoyl glycerols. *Phytochemistry.* 2012;76:25-31.

30. Cavin JF, Dartois V, Divies C: Gene cloning, transcriptional analysis, purification, and characterization of phenolic acid decarboxylase from *Bacillus subtilis*. *Appl Environ Microbiol.* 1998;64(4):1466-1471.

31. Guzik U, Hupert-Kocurek K, Sitnik M, Wojcieszynska D: Protocatechuate 3,4-dioxygenase: a wide substrate specificity enzyme isolated from *Stenotrophomonas maltophilia* KB2 as a useful tool in aromatic acid biodegradation. *Journal of molecular microbiology and biotechnology.* 2014;24(3):150-160.

32. Hammer A, Stolz A, Knackmuss H: Purification and characterization of a novel type of protocatechuate 3,4-dioxygenase with the ability to oxidize 4-sulfocatechol. *Archives of microbiology.* 1996;166(2):92-100.

33. Cavin JF, Barthelmebs L, Guzzo J, Beeumen J, Samyn B, Travers JF, Diviès C: Purification and characterization of an inducible *p* - coumaric acid decarboxylase from *Lactobacillus plantarum*. *FEMS Microbiol Lett.* 1997;147(2):291-295.

34. Ibdah M, Zhang XH, Schmidt J, Vogt T: A novel Mg^{2+}-dependent *O*-methyltransferase in the phenylpropanoid metabolism of *Mesembryanthemum crystallinum*. *The Journal of biological chemistry.* 2003;278(45):43961-43972.

35. Brand S, Hölscher D, Schierhorn A, Svatoš A, Schröder J, Schneider B: A type III polyketide synthase from *Wachendorfia thyrsiflora* and its role in diarylheptanoid and phenylphenalenone biosynthesis. *Planta.* 2006;224(2):413-428.

36. Schaefer AL, Greenberg EP, Oliver CM, Oda Y, Huang JJ, Bittan-Banin G, Peres CM, Schmidt S, Juhaszova K, Sufrin JR *et al*: A new class of homoserine lactone quorum-sensing signals. *Nature.* 2008;454(7204):595-599.

37. Weitzel C, Petersen M: Cloning and characterisation of rosmarinic acid synthase from *Melissa officinalis* L. *Phytochemistry.* 2011;72(7):572-578.

38. Luo J, Fuell C, Parr A, Hill L, Bailey P, Elliott K, Fairhurst SA, Martin C, Michael AJ: A novel polyamine acyltransferase responsible for the accumulation of spermidine conjugates in *Arabidopsis* seed. *Plant Cell.* 2009;21(1):318-333.

39. Kopycki JG, Stubbs MT, Brandt W, Hagemann M, Porzel A, Schmidt J, Schliemann W, Zenk MH, Vogt T: Functional and structural characterization of a cation-dependent *O*-methyltransferase from the cyanobacterium *Synechocystis* sp. strain PCC 6803. *The Journal of biological chemistry.* 2008;283(30):20888-20896.

40. Chapple CC, Vogt T, Ellis BE, Somerville CR: An *Arabidopsis* mutant defective in the general phenylpropanoid pathway. *Plant Cell.* 1992;4(11):1413-1424.

41. Dicko MH, Gruppen H, Hilhorst R, Voragen AG, van Berkel WJ: Biochemical characterization of the major sorghum grain peroxidase. *FEBS J.* 2006;273(10):2293-2307.

42. Melchior F, Kindl H: Grapevine stilbene synthase cDNA only slightly differing from chalcone synthase cDNA Is expressed in *Escherichia coli* into a catalytically active enzyme. *FEBS letters.* 1990;268(1):17-20.

43. Negrel J, Javelle F: Purification, characterization and partial amino acid sequencing of hydroxycinnamoyl-CoA:tyramine *N*-(hydroxycinnamoyl)transferase from tobacco cell-suspension cultures. *European journal of biochemistry / FEBS.* 1997;247(3):1127-1135.

44. Mavrides C, Orr W: Multispecific aspartate and aromatic amino acid aminotransferases in *Escherichia coli*. *The Journal of biological chemistry.* 1975;250(11):4128-4133.

45. Lim EK, Jackson RG, Bowles DJ: Identification and characterisation of *Arabidopsis* glycosyltransferases capable of glucosylating coniferyl aldehyde and sinapyl aldehyde. *FEBS letters.* 2005;579(13):2802-2806.

46. Lim EK, Li Y, Parr A, Jackson R, Ashford DA, Bowles DJ: Identification of glucosyltransferase genes involved in sinapate metabolism and lignin synthesis in *Arabidopsis*. *The Journal of biological chemistry.* 2001;276(6):4344-4349.

47. Gallage NJ, Hansen EH, Kannangara R, Olsen CE, Motawia MS, Jorgensen K, Holme I, Hebelstrup K, Grisoni M, Moller BL: Vanillin formation from ferulic acid in *Vanilla planifolia* is catalysed by a single enzyme. *Nat Commun.* 2014;5:4037.

48. Lindner HA, Nadeau G, Matte A, Michel G, Menard R, Cygler M: Site-directed mutagenesis of the active site region in the quinate/shikimate 5-dehydrogenase YdiB of *Escherichia coli*. *The Journal of biological chemistry.* 2005;280(8):7162-7169.

49. Fujiwara H, Tanaka Y, Fukui Y, Ashikari T, Yamaguchi M, Kusumi T: Purification and characterization of anthocyanin 3-aromatic acyltransferase from *Perilla frutescens*. *Plant Science.* 1998;137(1):87-94.

50. Nishimura M, Kohno K, Nishimura Y, Inagaki M, Davies J: Characterization of two isozymes of coniferyl alcohol dehydrogenase from *Streptomyces* sp. NL15-2K. *Biosci Biotechnol Biochem.* 2011;75(9):1770-1777.

51. Weitkamp P, Vosmann K, Weber N: Highly efficient preparation of lipophilic hydroxycinnamates by solvent-free lipase-catalyzed transesterification. *J Agric Food Chem.* 2006;54(19):7062-7068.

52. Stevenson DE, Parkar SG, Zhang JL, Stanley RA, Jensen DJ, Cooney JA: Combinatorial enzymic synthesis for functional testing of phenolic acid esters catalysed by *Candida antarctica* lipase B (Novozym 435 (R)). *Enzyme Microb Technol.* 2007;40(5):1078-1086.

53. Jang SM, Ishihara A, Back K: Production of coumaroylserotonin and feruloylserotonin in transgenic rice expressing pepper hydroxycinnamoyl-coenzyme A:serotonin *N*-(hydroxycinnamoyl)transferase. *Plant Physiol.* 2004;135(1):346-356.

54. Strack D, Gross W: Properties and activity changes of chlorogenic acid:glucaric acid caffeoyltransferase from Tomato (*Lycopersicon esculentum*). *Plant Physiol.* 1990;92(1):41-47.

55. Welle R, Grisebach H: Purification and properties of chalcone synthase from cell suspension cultures

of soybean. *Z Naturforsch C.* 1987;42(c):1200-1206.

56. Lin F, Ferguson KL, Boyer DR, Lin XN, Marsh EN: Isofunctional enzymes PAD1 and UbiX catalyze formation of a novel cofactor required by ferulic acid decarboxylase and 4-hydroxy-3-polyprenylbenzoic acid decarboxylase. *ACS Chem Biol.* 2015;10(4):1137-1144.

57. Anderson NA, Bonawitz ND, Nyffeler K, Chapple C: Loss of FERULATE 5-HYDROXYLASE leads to mediator-dependent inhibition of soluble phenylpropanoid biosynthesis in *Arabidopsis*. *Plant Physiol.* 2015;169(3):1557-1567.

58. Eudes A, Baidoo EE, Yang F, Burd H, Hadi MZ, Collins FW, Keasling JD, Loque D: Production of tranilast [*N*-(3',4'-dimethoxycinnamoyl)-anthranilic acid] and its analogs in yeast *Saccharomyces cerevisiae*. *Appl Microbiol Biotechnol.* 2011;89(4):989-1000.

59. Pereira JH, Heins RA, Gall DL, McAndrew RP, Deng K, Holland KC, Donohue TJ, Noguera DR, Simmons BA, Sale KL *et al*: Structural and biochemical characterization of the early and late enzymes in the lignin β-aryl ether cleavage pathway from *Sphingobium* sp. SYK-6. *J Biol Chem.* 2016;291(19):10228-10238.
