# Supplementary material for: Identification of enzymatic genes with the potential to reduce biomass recalcitrance through lignin manipulation in Arabidopsis
Source: Biotechnol Biofuels. 2020 May 29;13:97. doi: 10.1186/s13068-020-01736-6 (PMC7260809; doi:10.1186/s13068-020-01736-6)
Supplement: Supplementary file 2 — Additional file 2: Fig. S1. Histochemical GUS staining analysis. ß-glucuronidase (GUS) activity was monitored in transgenic Arabidopsis plants harboring a gene for GUS fused with PkC4Hpro (A, C, E, G, I, K, M, O, Q, S, U, and V). Lignin autofluorescence was also observed in each sample (B, D, F, H, J, L, N, P, R, and T). C, D, G, H, K, L, O, P, S, and T are the magnified views of A, B, E, F, I, J, M, N, Q, and R, respectively. Fig. S2. Wiesner staining of hand-cut sections. Samples were prepared from primary inflorescence stems of T1 plants harboring the single gene shown in each panel. Staining procedures were performed in 96-well plates. The diameter of each well was 7 mm. Bars indicate 150 µm. Fig. S3. Mäule staining of hand-cut sections. Samples were prepared from primary inflorescence stems of T1 plants harboring the single gene shown in each panel. Staining procedures were performed in 96-well plates. The diameter of each well was 7 mm. Bars indicate 150 µm. [file 13068_2020_1736_MOESM2_ESM.pptx]

## Slide 1
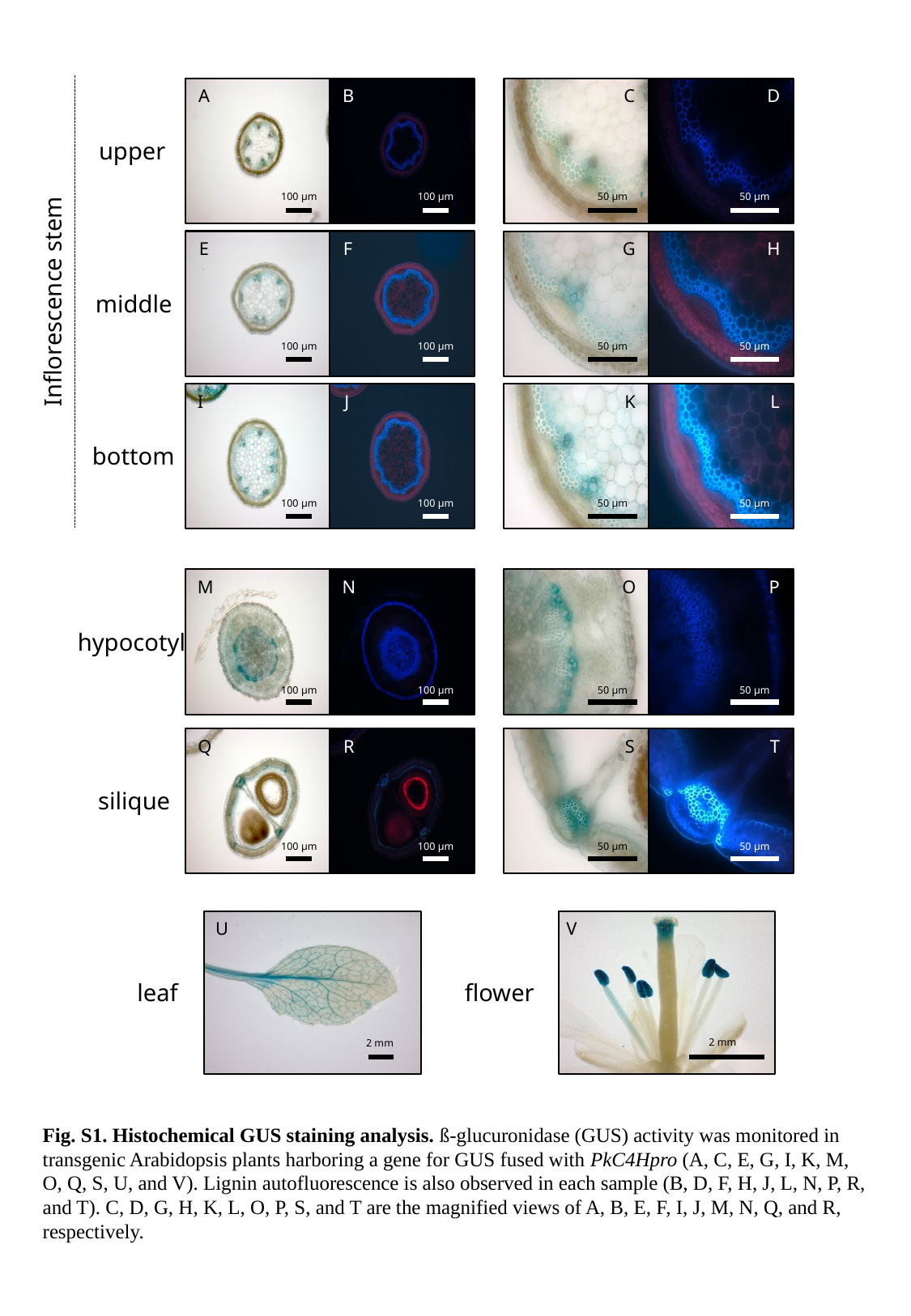

A
B
C
D
upper
100 μm
100 μm
50 μm
50 μm
E
F
G
H
Inflorescence stem
middle
100 μm
100 μm
50 μm
50 μm
I
J
K
L
bottom
100 μm
100 μm
50 μm
50 μm
M
N
O
P
hypocotyl
100 μm
100 μm
50 μm
50 μm
Q
R
S
T
silique
100 μm
100 μm
50 μm
50 μm
U
V
leaf
flower
2 mm
2 mm
Fig. S1. Histochemical GUS staining analysis. ß-glucuronidase (GUS) activity was monitored in transgenic Arabidopsis plants harboring a gene for GUS fused with PkC4Hpro (A, C, E, G, I, K, M, O, Q, S, U, and V). Lignin autofluorescence is also observed in each sample (B, D, F, H, J, L, N, P, R, and T). C, D, G, H, K, L, O, P, S, and T are the magnified views of A, B, E, F, I, J, M, N, Q, and R, respectively.

## Slide 2
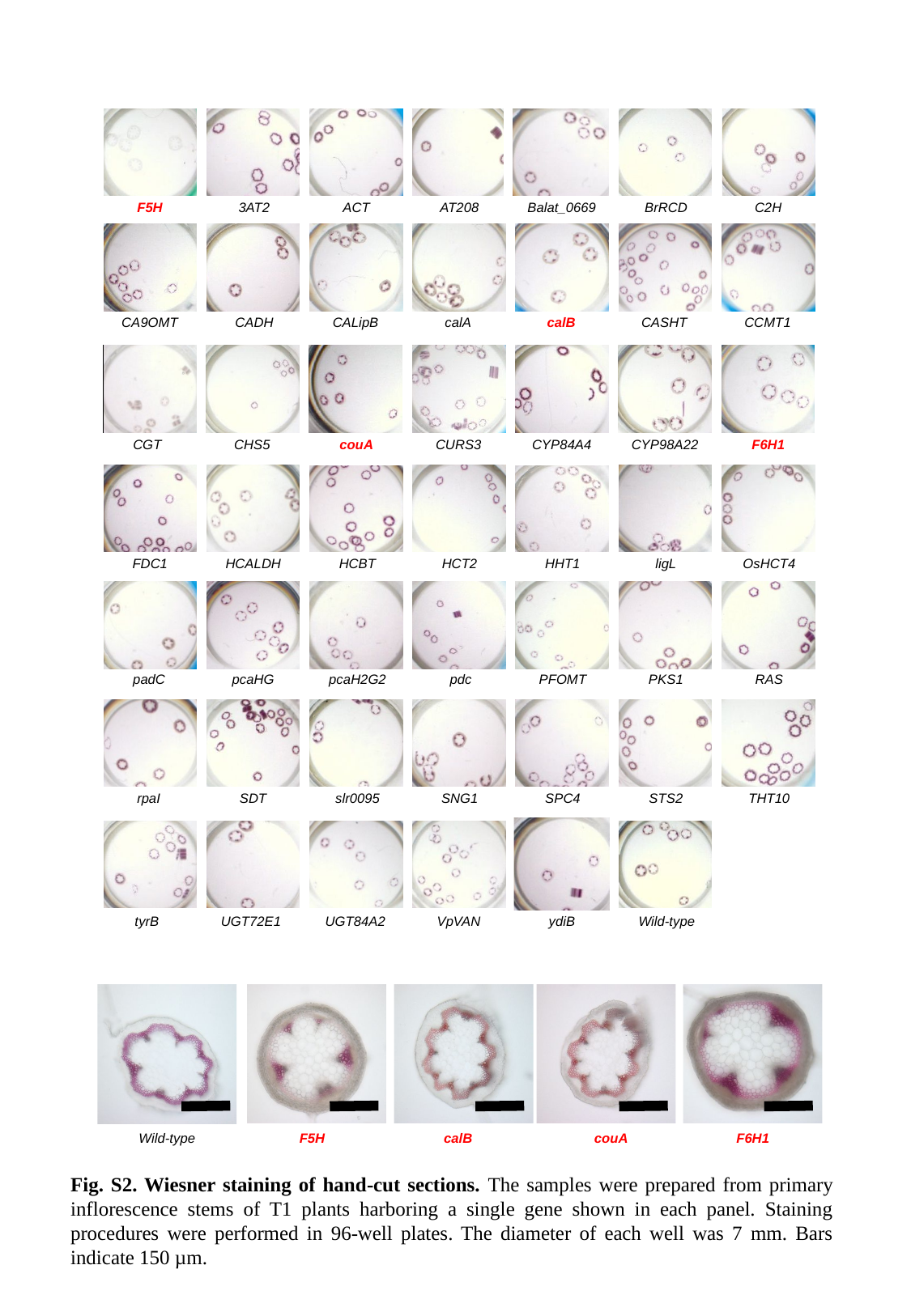

F5H
3AT2
ACT
AT208
Balat_0669
BrRCD
C2H
CA9OMT
CADH
CALipB
calA
calB
CASHT
CCMT1
CGT
CHS5
couA
CURS3
CYP84A4
CYP98A22
F6H1
FDC1
HCALDH
HCBT
HCT2
HHT1
ligL
OsHCT4
padC
pcaHG
pcaH2G2
pdc
PFOMT
PKS1
RAS
rpaI
SDT
slr0095
SNG1
SPC4
STS2
THT10
tyrB
UGT72E1
UGT84A2
VpVAN
ydiB
Wild-type
Wild-type
F5H
calB
couA
F6H1
Fig. S2. Wiesner staining of hand-cut sections. The samples were prepared from primary inflorescence stems of T1 plants harboring a single gene shown in each panel. Staining procedures were performed in 96-well plates. The diameter of each well was 7 mm. Bars indicate 150 µm.

## Slide 3
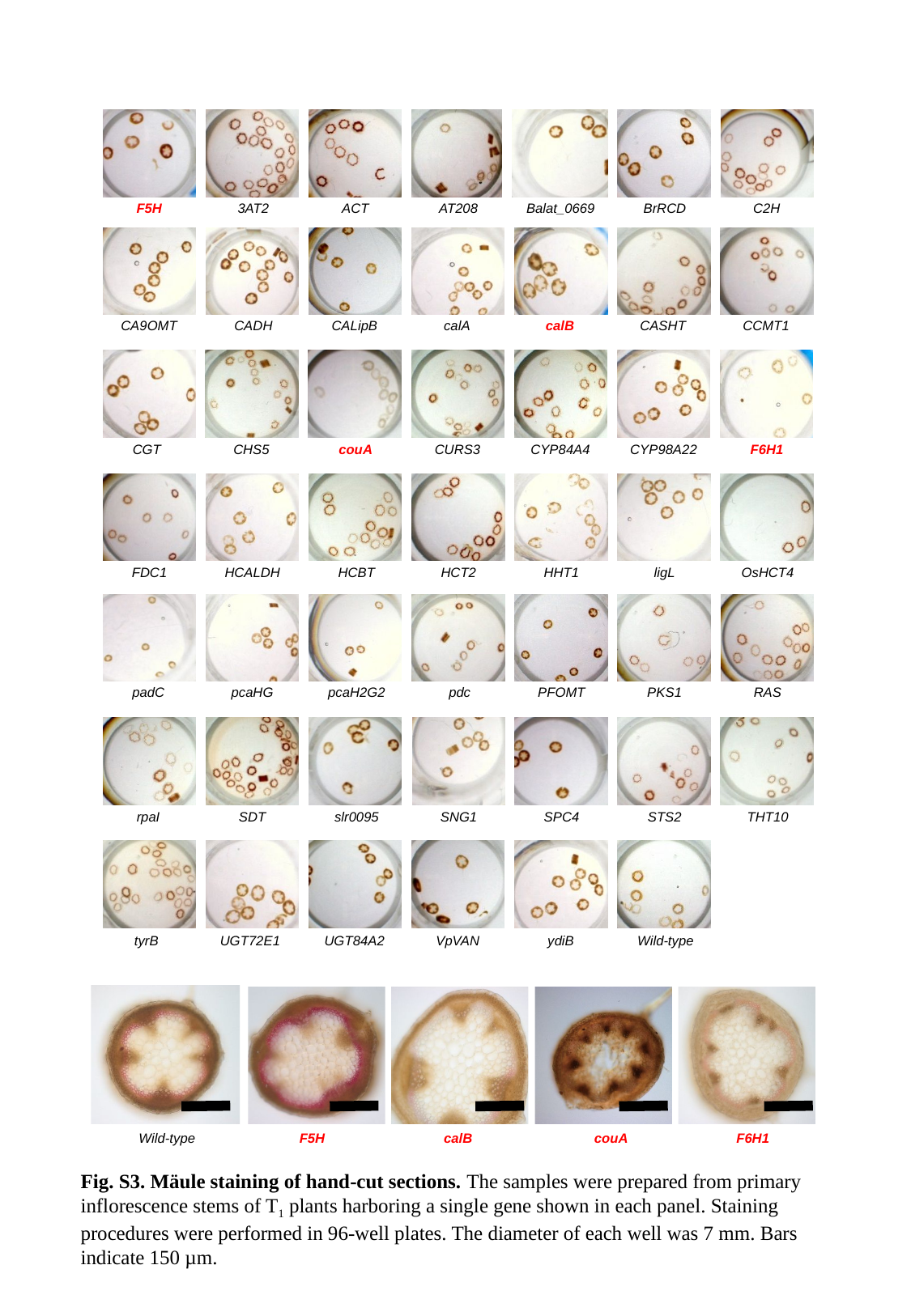

F5H
3AT2
ACT
AT208
Balat_0669
BrRCD
C2H
CA9OMT
CADH
CALipB
calA
calB
CASHT
CCMT1
CGT
CHS5
couA
CURS3
CYP84A4
CYP98A22
F6H1
FDC1
HCALDH
HCBT
HCT2
HHT1
ligL
OsHCT4
padC
pcaHG
pcaH2G2
pdc
PFOMT
PKS1
RAS
rpaI
SDT
slr0095
SNG1
SPC4
STS2
THT10
tyrB
UGT72E1
UGT84A2
VpVAN
ydiB
Wild-type
Wild-type
F5H
calB
couA
F6H1
Fig. S3. Mäule staining of hand-cut sections. The samples were prepared from primary inflorescence stems of T1 plants harboring a single gene shown in each panel. Staining procedures were performed in 96-well plates. The diameter of each well was 7 mm. Bars indicate 150 µm.
